# Local differentiation amidst extensive allele sharing in *Oryza nivara* and *O. rufipogon*

**DOI:** 10.1002/ece3.689

**Published:** 2013-08-01

**Authors:** Maria Celeste N Banaticla-Hilario, Ronald G van den Berg, Nigel Ruaraidh Sackville Hamilton, Kenneth L McNally

**Affiliations:** 1 T.T. Chang Genetic Resources Center, International Rice Research InstituteLos Baños, Laguna, Philippines; 2 Biosystematics Group, Wageningen University and Research CenterWageningen, The Netherlands

**Keywords:** Species divergence, SSR diversity, sympatric populations, wild *Oryza*

## Abstract

Genetic variation patterns within and between species may change along geographic gradients and at different spatial scales. This was revealed by microsatellite data at 29 loci obtained from 119 accessions of three *Oryza* series *Sativae* species in Asia Pacific: *Oryza nivara* Sharma and Shastry*, O. rufipogon* Griff., and *O. meridionalis* Ng. Genetic similarities between *O. nivara* and *O. rufipogon* across their distribution are evident in the clustering and ordination results and in the large proportion of shared alleles between these taxa. However, local-level species separation is recognized by Bayesian clustering and neighbor-joining analyses. At the regional scale, the two species seem more differentiated in South Asia than in Southeast Asia as revealed by *F*_ST_ analysis. The presence of strong gene flow barriers in smaller spatial units is also suggested in the analysis of molecular variance (AMOVA) results where 64% of the genetic variation is contained among populations (as compared to 26% within populations and 10% among species). *Oryza nivara* (*H*_E_ = 0.67) exhibits slightly lower diversity and greater population differentiation than *O. rufipogon* (*H*_E_ = 0.70). Bayesian inference identified four, and at a finer structural level eight, genetically distinct population groups that correspond to geographic populations within the three taxa. *Oryza meridionalis* and the Nepalese *O. nivara* seemed diverged from all the population groups of the series, whereas the Australasian *O. rufipogon* appeared distinct from the rest of the species.

## Introduction

The unwavering pursuit to fully understand the rice gene pool is reflected in the growing number of publications on *Oryza rufipogon* and *O. nivara*. These two closest relatives of Asian cultivated rice (*O. sativa* L.) are morphologically distinct (Ng et al. 1981; Uga et al. 2003; Banaticla-Hilario 2012), whereas genetic isolation is effected by differences in habitat, mating system, and flowering time. Their geographic distributions show overlap in tropical continental Asia with *O. rufipogon* extending southeastward to insular Southeast Asia and Australasia.

*Oryza nivara* is variously treated as a distinct species (Sharma and Shastry 1965; Ng et al. 1981; Lu 1999; Lu et al. 2001) or as an ecotype of *O. rufipogon* (Tateoka 1963; Oka 1988; Vaughan et al. 2003). This taxonomic ambivalence is also reflected in incongruencies between the results of different molecular data, where isozymes (Second 1985), random amplification of polymorphic DNAs (Ren et al. 2003), allozymes and restriction fragment length polymorphisms (Cai et al. 2004), transposon display markers (Kwon et al. 2006), tourist sequences (Iwamoto et al. 1999), miniature inverted-repeat transposable elements in amplified fragment length polymorphisms (Park et al. 2003), microsatellites (Ren et al. 2003), sequence tagged sites (Huang et al. 2012a), and various genes sequences (Zhu and Ge 2005; Zhou et al. 2008; Zheng and Ge 2010) did not detect divergence between *O. nivara* and *O. rufipogon,* whereas AFLPs (Aggarwal et al. 1999), microsatellites (Kuroda et al. 2007; Singh et al. 2013), combined sequences from chloroplast, mitochondrial and nuclear DNA (Duan et al. 2007), and single nucleotide polymorphisms (SNPs) (Xu et al. 2012) did indicate a separation at species level. In this study, the annual taxon is tentatively considered as a distinct species.

Genetic differentiation between *O. nivara* and *O. rufipogon* has been examined globally using populations sampled across the species' total geographic distribution (Zheng and Ge 2010; Huang et al. 2012a) and at a regional scale by comparing patterns in South and Southeast Asia (Lu et al. 2008). Local-scale studies were conducted by Kuroda et al. (2007) and Singh et al. (2013) using Lao and Indian populations, respectively. However, spatial patterns of intra- and interspecific differentiation remain unclear for these two taxa.

The closely related taxon, *O. meridionalis*, is a genetically distinct species (Xu et al. 2005; Kwon et al. 2006) that exhibits a similar life cycle, breeding habit, phenology, and habitat to that of *O. nivara,* but geographically overlaps only with the southern limit of *O. rufipogon*. Therefore, it seems worthwhile to compare the genetic differences between *O. meridionalis* and *O. rufipogon* with those between *O. nivara* and *O. rufipogon*.

A number of questions on the relationship between *O. nivara* and *O. rufipogon* remain: Are the observed genetic similarities/differences consistent along spatial gradients and across varying geographical units? Are locally sympatric populations of *O. nivara* and *O. rufipogon* more differentiated than the nonsympatric ones? How does geography influence the variations within and between the three *Oryza* species?

In an effort to answer these questions and uncover underlying spatial variation patterns, this study analyzes locally sympatric accession pairs (i.e., populations of different species collected from the same locality) of *O. nivara* and *O. rufipogon* from across South Asia and continental Southeast Asia and of *O. meridionalis* and *O. rufipogon* in Australasia (New Guinea and Australia) as well as *O. rufipogon* populations from insular Southeast Asia. These three taxa, along with cultivated rice compose *Oryza* series *Sativae* in the Asia-Pacific area.

For this study we use simple sequence repeat (SSR) markers to: (1) determine global-, regional-, and local-scale differentiation between *O. nivara* and *O. rufipogon*; (2) infer geographic population groups in Asia-Pacific *Oryza* series *Sativae*; and (3) assess genetic diversity at the population group and species level.

## Material and Methods

### Plant material

One hundred nineteen accessions from the International Rice Genebank (IRG) at the International Rice Research Institute (IRRI) in the Philippines were selected to represent sympatric populations of *O. nivara* and *O. rufipogon* and of *O. meridionalis* and *O. rufipogon* across their distribution range, as well as *O. rufipogon* populations that are nonsympatric to both annual species (Table S1). Due to limited availability and germination issues, only one accession from China was sampled. The same plant material was used in a previous phenotyping experiment (Banaticla-Hilario 2012) wherein some accessions were tentatively classified as intermediate forms (i.e., intermediate between two wild species or between *O. sativa* and a wild species) (Table S1). We included these intermediate forms in this study to determine their genetic affinity with the other *Oryza* series *Sativae* species in Asia. IRGC 81837, 89228, and 106152 displayed two different plant types within the accession and were thus represented as two separate subpopulations (N26A and N26B, R5A and R5B, and R29A and R29B, respectively). Six *O. sativa* accessions were also included for comparison (Table S1). The plant material was grown in the Genetic Resources Center screenhouse at IRRI, the Philippines. Leaf samples were harvested from five individual plants per accession.

Genomic DNA was extracted from fresh leaf samples by applying the modified CTAB (cetyl trimethyl ammonium bromide) extraction protocol (Fulton et al. 1995). The DNA samples were quantified using spectrophotometry (NanoDrop™ 1000 spectrophotometer, Thermo Fisher Scientific, Wilmington, DE) and gel densitometry (using Lambda DNA as a standard), and then normalized to 5 ng/μL concentration.

### SSR genotyping

The markers used (Table 1) were from the panel of 30 standard SSR markers developed by the Generation Challenge Program for rice diversity analysis (http://gramene.org/markers/microsat/50_ssr.html). However, RM514 did not amplify well in most of the samples and was dropped from the analysis.

**Table 1:** Basic information and overall diversity of the 29 SSR markers used in the study

Marker	Chr	*Oryza sativa* varietal group (germplasm)	Motif	AT (°C)	A_N_	RA_N_	MA_F_	PIC
RM237	1	indica (IR36)	(CT)18	55	23	17	0.1860	0.9056
RM283	1	indica (IR36)	(GA)18	61	20	13	0.2140	0.8869
RM431	1	japonica (Nipponbare)	(AG)16	55	19	11	0.1280	0.8925
RM495	1	japonica (Nipponbare)	(CTG)7	55	4	0	0.7480	0.3870
RM154	2	unknown	(GA)21	61	24	19	0.2480	0.8416
RM452	2	japonica (Nipponbare)	(GTC)9	61	9	5	0.7500	0.3985
OSR13	3	unknown	(GA)n	53	19	12	0.2080	0.8783
RM338	3	indica (IR36)	(CTT)6	55	4	2	0.9280	0.1293
RM124	4	japonica (Nipponbare)	(TC)10	67	9	6	0.7100	0.4477
RM161	5	japonica (Nipponbare)	(AG)20	61	17	11	0.2570	0.8442
RM413	5	japonica (Nipponbare)	(AG)11	53	22	15	0.2580	0.8745
RM507	5	japonica (Nipponbare)	(AAGA)7	55	7	4	0.7320	0.3984
RM133	6	japonica (Nipponbare)	(CT)8	63	7	3	0.4470	0.6093
RM162	6	unknown	(AC)20	61	22	15	0.2790	0.8467
RM118	7	japonica (Nipponbare)	(GA)8	67	9	6	0.4000	0.6216
RM125	7	japonica (Nipponbare)	(GCT)8	63	13	6	0.2420	0.8362
RM455	7	japonica (Nipponbare)	(TTCT)5	57	3	1	0.8970	0.1691
RM44	8	indica (IR36)	(GA)16	53	18	7	0.1690	0.8897
RM152	8	unknown	(GGC)10	53	9	5	0.4450	0.6453
RM408	8	japonica (Nipponbare)	(CT)13	55	11	8	0.4150	0.6948
RM447	8	japonica (Nipponbare)	(CTT)8	55	15	8	0.1610	0.8723
RM284	8	indica (IR36)	(GA)8	55	10	4	0.5990	0.5447
RM433	8	japonica (Nipponbare)	(AG)13	53	18	11	0.2030	0.8591
RM215	9	indica (IR36)	(CT)16	55	21	13	0.1460	0.8904
RM316	9	indica (IR36)	(GT)8-(TG)9(TTTG)4(TG)4	55	32	28	0.1900	0.9048
RM271	10	indica (IR36)	(GA)15	55	23	14	0.1590	0.9135
RM484	10	japonica (Nipponbare)	(AT)9	55	8	3	0.3080	0.7381
RM536	11	japonica (Nipponbare)	(CT)16	55	12	7	0.3540	0.7064
RM277	12	indica (IR36)	(GA)11	55	9	5	0.3510	0.6941
Mean					14	9	0.3909	0.6934

Chr, chromosome number; AT, annealing temperature; A_N_, number of alleles; RA_N_, number of rare alleles (allele frequency ≤5%); MA_F_, frequency of major allele; PIC, polymorphism information content.

Polymerase chain reaction (PCR) was conducted in 20 μL reaction volume composed of 5.92 μL sterilized ultrapure water, 2 μL each of 10× MgCl_2_ free buffer, 10 mmol/L deoxynucleotide triphosphates, and 25 mmol/L MgCl_2_ (iNtRON Biotechnology, Kyungki-Do, South Korea), 0.08 μL of 5U/μL *i-Taq*™ DNA polymerase (iNtRON Biotechnology), 1 μL of 1 μmol/L labeled M13 forward primer (IRDye 700 or 800, LI-COR Biosciences, Lincoln, NE), 1 μL of 1 μmol/L M13-tailed SSR forward primer (Invitrogen, Carlsbad, CA), 2 μL of 1 μmol/L SSR reverse primer (Invitrogen), and 4 μL of genomic DNA. The program started with denaturation at 95°C for 2 min, succeeded by 32 cycles of denaturation at 95°C (30 sec), annealing at 55°C (30 sec) and elongation at 72°C (50 sec), then followed by a 2 min final extension step at 72°C. The annealing temperature was adjusted to match the optimal value for each marker as indicated in Table 1.

PCR products were multiplexed by combining 2 μL each of IRDye 700- and IRDye 800-labeled samples, 5 μL sterilized nanopure water, and 5 μL loading dye. Gel electrophoresis was performed on the 4300 LI-COR DNA analyzer system. The LI-COR IRDye 50–350 bp size standard ladder was used to estimate allele size. The gels were analyzed and scored with the SAGA Generation 2 software (LI-COR, Biosciences, Lincoln, NE).

### Data analyses

Genetic diversity measures were estimated for markers, populations, inferred population groups, and species. The number of alleles and rare alleles (frequency ≤5%), frequency of major allele (allele with the highest frequency), observed heterozygosity, unbiased estimate of gene diversity (Weir 1996), polymorphism information content (PIC), and inbreeding coefficient (*F*_IS_) were obtained using PowerMarker 3.25 (Liu and Muse 2005). Allelic richness values were determined with FSTAT (Goudet 2001).

A cluster analysis was conducted with PowerMarker 3.25 (Liu and Muse 2005). A neighbor-joining (NJ) tree based on C.S. Chord distance (Cavalli-Sforza and Edwards 1967) was constructed and bootstrapping with 1000 replicates was performed. The output trees were viewed and edited with MEGA 5.05 (Tamura et al. 2011).

Principal coordinate analysis (PCoA) of C.S. Chord distance matrices between species across distribution was performed with GenAlEx 6.4 (Peakall and Smouse 2006).

Genetically distinct populations were inferred by applying the nonspatial and spatially explicit Bayesian clustering algorithms of STRUCTURE 2.2 (Pritchard et al. 2000; Falush et al. 2003) and TESS 2.3.1 (Durand et al. 2009), respectively.

A STRUCTURE model allowing for admixture and assuming correlated allele frequencies was implemented. Cluster values ranging from *K* = 1 to *K* = 15 were tested with 20 independent runs for each *K* with a burn-in of 20,000 iterations and run length of 20,000 iterations per run. The 10 runs with the highest posterior probability ln *P(D)* values were selected from each *K* and their average ln *P(D)* were used in calculating the delta *K* (Δ*K*), a statistic based on the rate of changes in the likelihood distribution between successive *K* values (Evanno et al. 2005). The cluster value corresponding to the highest peak in the Δ*K* plot is considered as the appropriate cluster solution.

As the TESS program uses spatial prior information, the geographically underrepresented set of *O. sativa* accessions were excluded from the analysis. Misidentified accessions detected by NJ, PCoA, and STRUCTURE analysis were also removed from the runs. Prior to analysis, the “generate spatial coordinates” option of TESS was used to create individual sample coordinates based on accession coordinates. In the TESS runs, the conditional autoregressive (CAR) model of admixture (admixture parameter = 1.0, spatial interaction parameter = 0.6) was implemented with a linear trend surface. The maximal number of clusters was set to range from *K*_max_ = 2 to *K*_max_ = 10. Each *K*_max_ was tested with 100 runs, and each run had 20,000 burn-in sweeps followed by another 30,000 sweeps. To determine the appropriate number of TESS clusters, the deviance information criterion (DIC) should be analyzed and the stability of the bar plots should be considered (Durand et al. 2009). From each *K*_max_, the 10 runs with the lowest DIC were selected and their mean DIC values were plotted against *K*_max_. The optimum cluster solution is the *K*_max_ value that coincides with the plateau of the DIC curve.

For each *K*/*K*_max_ (from *K* = 2 to *K* = 8), the 10 STRUCTURE runs with maximal ln (*P*[*D*]) values as well as the 10 TESS runs with the lowest DIC values were aligned and averaged using CLUMPP 1.1.2 (Jakobsson and Rosenberg 2007), employing the Greedy algorithm with 10,000 random permutations (except for *K* = 8 where the LargeKGreedy algorithm with 10,000 random permutations was used). The output files (bar graphs) were viewed and edited with the DISTRUCT software (Rosenberg 2004).

GenAlEx 6.4 (Peakall and Smouse 2006) was used in conducting an analysis of molecular variance (AMOVA) and in estimating pairwise *F*_ST_ values between inferred population groups.

## Results

### Overall microsatellite diversity in Asia-Pacific *Oryza* series *Sativae*

Across the 119 *Oryza* series *Sativae* populations from Asia Pacific, 417 alleles (62% of which are rare) were detected at the 29 SSR loci. The number of alleles per locus varied from three (RM455) to 32 (RM316), with an average of 14. The number of rare alleles ranged from zero (RM495) to 28 (RM316), with an average of nine. The most common allele in each locus had a mean frequency of 0.39 and varied from 0.13 (RM431) to 0.93 (RM338). The PIC values differed from 0.13 (RM338) to 0.91 (RM237 and RM271) and had a mean value of 0.69 (Table 1). Based on the mentioned parameters, the most diverse loci were RM154, RM271, RM237, and RM316, whereas the least diverse were RM338, RM455, and RM495.

### Cluster analysis

**Figure 1:**
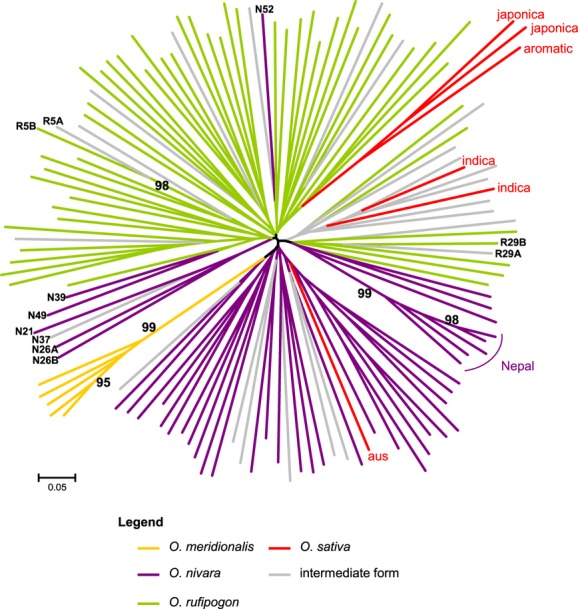
NJ tree of *Oryza* series *Sativae* populations based on C.S. Chord distance. Bootstrap values above 50% are displayed at the root of the supported cluster. The populations are colored according to their morphological classification.

The NJ tree (Fig. 1) shows the general tendency of accessions from the same species to cluster together. An admixed cluster composed of eight *O. nivara*, seven *O. rufipogon*, two *O. sativa* var. *indica*, and nine phenotypically intermediate accessions was also produced. However, the *O. nivara* and *O. rufipogon* clusters as well as the admixed cluster have very low bootstrap values (less than 50%) and only the *O. meridionalis* and Nepalese *O. nivara* clusters have strong bootstrap support (both with 99% bootstrap value). *Oryza nivara* and *O. meridionalis* join in a large cluster where the latter forms a distinct group that seems closer to the Southeast Asian populations of the former. The Nepalese *O. nivara* forms a separate branch within the admixed cluster. The aromatic and japonica populations of *O. sativa* group with several South Asian accessions of *O. rufipogon,* whereas the Australasian population joins a cluster composed of both South and Southeast Asian *O. nivara*. Three *O. nivara* populations from India (N21, N26A, and N26B) and an intermediate population from Sri Lanka (N37) separated from the annual species and form a cluster. Three *O. nivara* accessions (N39, N49, and N52) group with *O. rufipogon* while the intermediate populations are distributed among different clusters.

### Principal coordinate analysis

**Figure 2:**
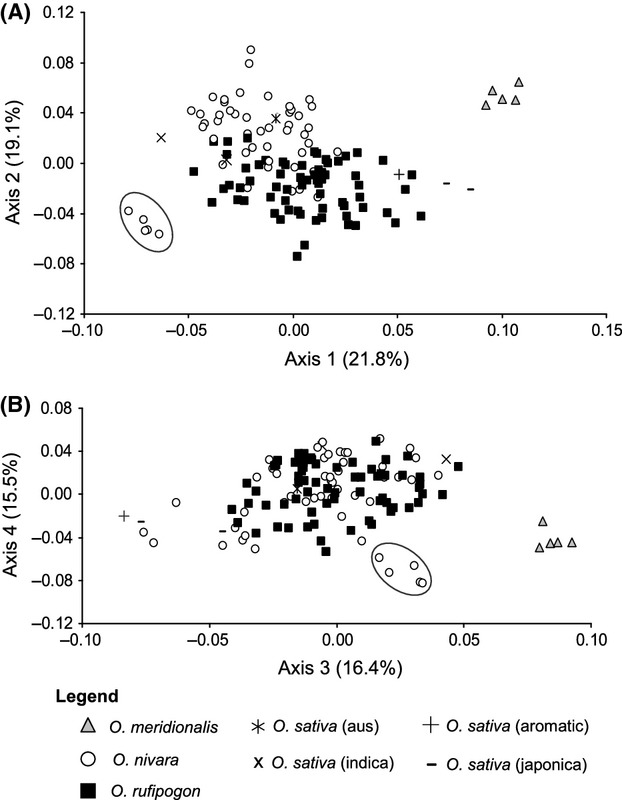
Principal coordinate plots revealing the genetic distinctiveness among *Oryza rufipogon, O. meridionalis, O. nivara,* and *O. sativa* accessions. *Oryza nivara* accessions from Nepal are encircled. (A) Axis 1 versus axis 2. (B) Axis 3 versus axis 4.

The first two principal coordinate axes reflect separate but partially overlapping clusters of *O. nivara* and *O. rufipogon* (Fig. 2A). *Oryza meridionalis* and Nepalese *O. nivara* accessions form distinct clusters isolated by axes 1 (21.83% proportion of variance) and 2 (19.10%). *Oryza sativa* accessions are distributed throughout the plot with aromatic and japonica populations joining *O. rufipogon,* Australasian, and one indica accession grouping with *O. nivara*, and the other indica accession in the middle of the *O. nivara* – *O. rufipogon* complex (Fig. 2A). The third and fourth principal coordinate axes do not separate *O. nivara* from *O. rufipogon* (Fig. 2B). Axis 3 (16.43%) isolates *O. meridionalis,* whereas axis 4 (15.52%) separates the Nepalese *O. nivara* from the rest of the taxa. The succeeding principal coordinate axes displayed uninformative clustering patterns.

### Bayesian clustering

TESS exhibits more consistent runs and produces more stable population clusters (with less fragmented members) than STRUCTURE as indicated in the average membership coefficients of each *K* (from *K* = 2 to *K* = 8) obtained by CLUMPP (Fig. S1). Across different *K* values in the STRUCTURE runs, populations N39 and N49 cluster with *O. rufipogon,* whereas R10, R43, and R50 are grouped with *O. nivara*. These apparently mislabelled populations also do not cluster with their supposed species groups in the NJ and PCA results and were excluded from the TESS runs.

**Figure 3:**
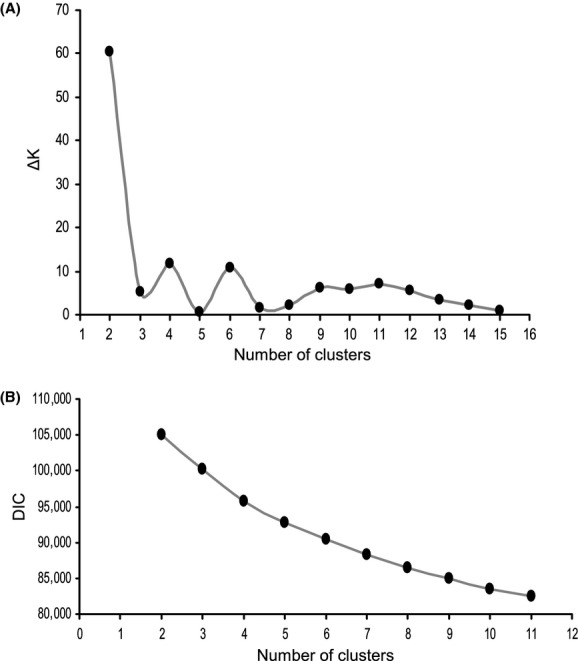
Criteria used in determining the appropriate cluster solution: (A) Delta *K* plot of STRUCTURE runs based on ln *P(D)* values; and (B) Plot of the mean DIC value of each TESS cluster solution. Plot lines were added to help visualize trends.

**Figure 4:**
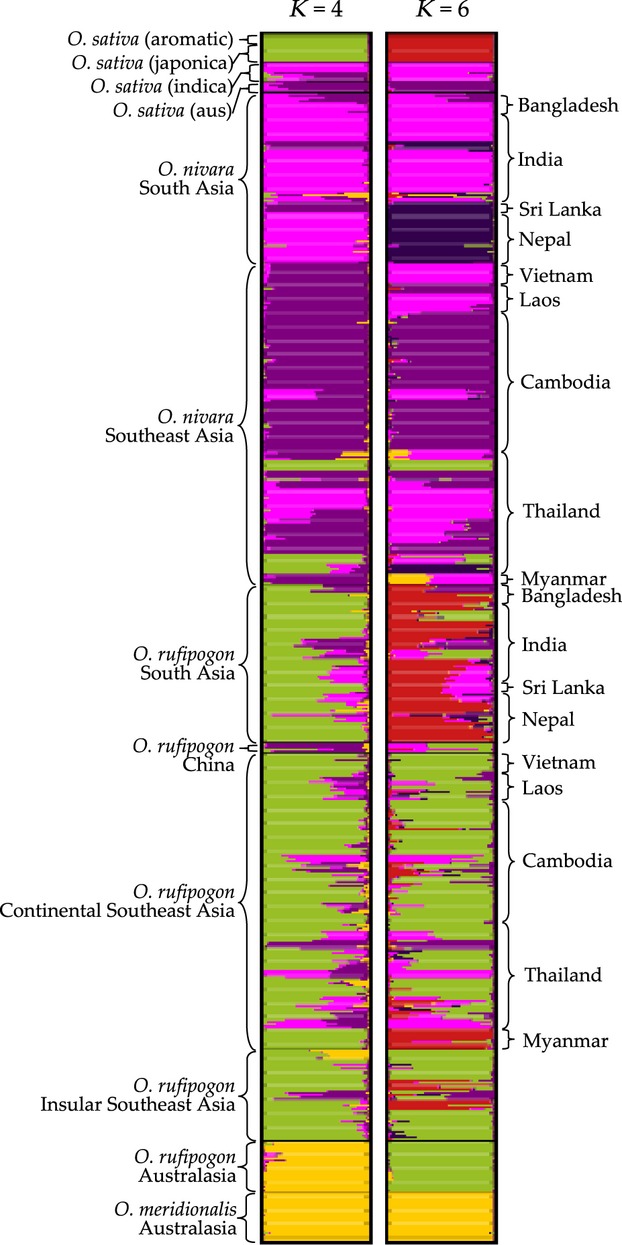
Population clusters of STRUCTURE at *K* = 4 and *K* = 6 (based on the modal clustering pattern). The predefined population assignments are on the left-hand side and the geographic origin of sympatric populations are on the right-hand side of the figure.

**Figure 5:**
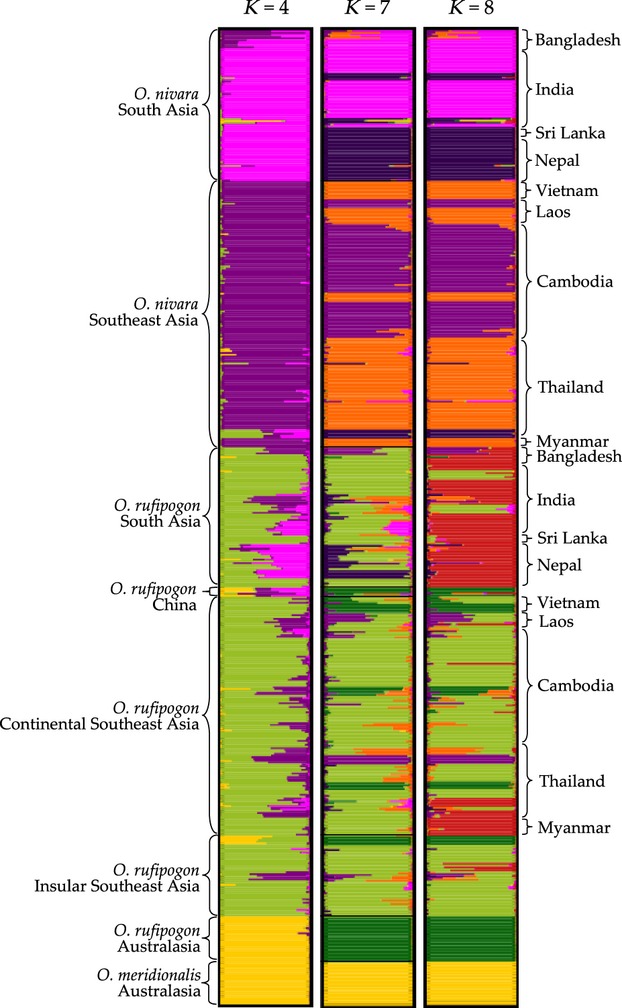
Population clusters of TESS at *K* = 4, *K* = 7, and *K* = 8 (based on the modal clustering pattern). The predefined population assignments are on the left-hand side and the geographic origin of sympatric populations are on the right-hand side of the figure.

The Δ*K* plot of the STRUCTURE runs displays distinct peaks at *K* = 2 (the highest value), *K* = 4, and *K* = 6 (Fig. 3A). However, *K* = 2 is rejected as an optimal cluster value as the cluster solution produced by STRUCTURE fails to distinguish *O. meridionalis* as a distinct population (Fig. S1). The relatively stable membership coefficient plots of both STRUCTURE and TESS runs at *K* = 4 (Fig. S1) suggest that four clusters optimally define the population structure of the data set. The four population groups depicted similarly by STRUCTURE and TESS are as follows: (C1) South Asian *O. nivara*; (C2) Southeast Asian *O. nivara*; (C3) continental and insular Asian *O. rufipogon*; and (C4) *O. meridionalis* and Australasian *O. rufipogon* (Figs. 4, 5). However, at *K* = 4, certain populations are occasionally swapped between different clusters across the 10 STRUCTURE runs (Fig. S2). Australasian *O. rufipogon* frequently joins *O. meridionalis* but also groups with either the rest of *O. rufipogon* or the japonica group of *O. sativa* in the other runs. The Nepalese and the Indian–Bangladeshi populations of *O. nivara* cluster together once, but in the rest of the runs, the former groups with either *O. rufipogon* or *O. meridionalis* while the latter joins either the Southeast Asian *O. nivara* or *O. meridionalis* (Fig. S2). The 10 TESS runs show more consistent population clustering at *K* = 4, with eight runs grouping the Australasian *O. rufipogon* with *O. meridionalis* and nine runs splitting *O. nivara* into South Asian and Southeast Asian populations (Fig. S3B).

At *K* = 6, the groups recognized by STRUCTURE are as follows (Fig. S2; Fig. 4): an *O. meridionalis* cluster (in 70% of the runs); two clusters in *O. rufipogon* (a South Asian group joined by the aromatic and japonica accessions of *O. sativa* and a Southeast Asian cluster in 40% of the runs); and three clusters in *O. nivara* (one cluster is predominantly Cambodian and groups with *O. sativa* aus, another cluster is mainly Nepalese, and the third cluster comprises the rest of *O. nivara* and is grouped with *O. sativa* indica, in 20% of the runs). Nevertheless, the output of the six-cluster solution of STRUCTURE (and even TESS) seems unstable because certain populations (particularly, the Australasian *O. rufipogon* and the non-Nepalese South Asian *O. nivara*) appear fragmented and/or are swapped between different clusters (Figs. S2, S3C).

**Figure 6:**
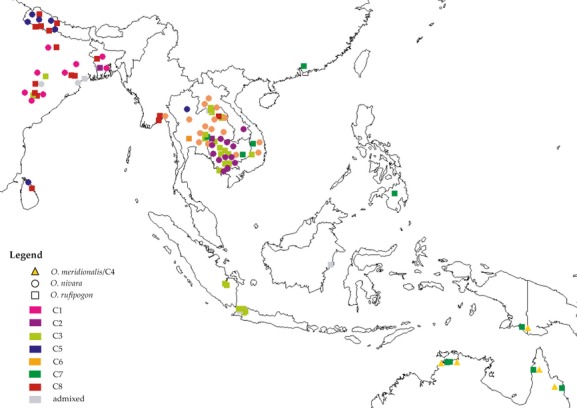
Distribution map of the eight population groups of Asia-Pacific *Oryza* series *Sativae* inferred by TESS. The geographic coordinates were slightly adjusted to allow better graphical representation of overlapping accessions.

The DIC plot of the TESS runs does not exhibit a well-defined plateau as the DIC values continuously decrease at higher *K*_max_ (Fig. 3B). Across the 10 TESS runs, *K* = 8 shows the most consistent grouping of populations (Figs. S1, S3D). Moreover, higher *K*_max_ values (*K* = 9 and *K* = 10), display less stable clustering and do not recognize additional distinct population clusters aside from the groups inferred at *K* = 8 (Fig. S3D and E). This indicates that the eight-cluster solution fits the lower population structure level of the data set. *K* = 4 and *K* = 7 also produce stable bar plots (Figs. S1, S3B and C) and will be discussed for comparison purposes. The clustering pattern of *K* = 4 was discussed previously with the STRUCTURE results. At *K* = 7, the inferred groups are as follows: (C1) Indian and Bangladeshi *O. nivara*; (C2) Cambodian *O. nivara*; (C3) continental and insular Asian *O. rufipogon*; (C4) *O. meridionalis*; (C5) Nepalese *O. nivara*; (C6) non-Cambodian *O. nivara*; and (C7) Australasian *O. rufipogon* (Fig. 5). At *K* = 8, the same population groups are recognized except for C3 (Asian *O. rufipogon*) that is split into the Southeast Asian *O. rufipogon* (C3 of *K* = 8) and South Asian *O. rufipogon* (C8 of *K* = 8) clusters (Fig. 5). The geographic subdivisions in *O. nivara* and *O. rufipogon* are illustrated in the distribution map of the eight population clusters (Fig. 6), where the local separation of the two species across their range is also depicted.

Following Garris et al. (2005) and Agrama et al. (2010), a cut-off membership coefficient (Table S2) of ≥0.6 was imposed to assign each population to a cluster. The relationships between population clusters at *K* = 4, *K* = 7, and *K* = 8 are depicted by the PCoA plots in Figures S4, S5. The fourth inferred group (C4 – Australasian *O. rufipogon* and *O. meridionalis*) at *K* = 4 seems dubious as the genetic uniqueness of *O. meridionalis* detected by PCoA is not recognized in the said cluster solution (Figs. S4A, S5A). At *K* = 7 and *K* = 8, C1, C2, and C6 form the *O. nivara* cluster and C3, C7, and C8 (for *K* = 8) comprise the *O. rufipogon* group (Fig. S4B and C), whereas C4 (*O. meridionalis*) and C5 (Nepalese *O. nivara*) form distinct clusters (Figs. S4B and C, S5B and C). Principal coordinate axis 1 separates C7 (Australasian *O. rufipogon*) from the rest of *O. rufipogon* at *K* = 7 and *K* = 8 (Fig. S4B and C), whereas axis 4 separates the South Asian clusters (C1, C5, and C8) from the Southeast Asian groups (C2, C3, and C6) at *K* = 8 (Fig. S5C).

### Genetic diversity of species and population groups

The annual species and population groups (*O. meridionalis* and *O. nivara*) exhibit higher *F*_IS_ values (0.94–0.98) than the perennial taxa (0.85–0.87) (Table 2). Among the three species, *O. rufipogon* contains the largest genetic variation as it displays the highest values in all diversity parameters. In contrast, *O. meridionalis* has the lowest values, rendering it the least diverse species and population group.

**Table 2:** Microsatellite diversity of species and population groups in Asia-Pacific *Oryza* series *Sativae* (based on 29 SSR loci)

Taxon (TESS cluster number)	S	A_L_	RA_L_	R_S_	*H*_O_	*H*_E_	PIC	*F*_IS_
Species								
*O. nivara*	229	9.83	5.03	7.47	0.02	0.67	0.65	0.97
*O. rufipogon*	226	11.79	6.72	8.43	0.09	0.70	0.68	0.87
*O. meridionalis* (C4)	24	2.24	0.21	2.24	0.01	0.24	0.23	0.98
Population clusters								
Indian and Bangladeshi *O. nivara* (C1)	46	4.28	0.86	4.06	0.02	0.52	0.48	0.97
Nepalese *O. nivara* (C5)	35	3.59	0.76	3.47	0.02	0.39	0.37	0.94
Cambodian *O. nivara* (C2)	72	6.55	2.31	5.70	0.03	0.59	0.56	0.95
Non-Cambodian *O. nivara* (C6)	73	6.48	1.59	5.88	0.02	0.61	0.59	0.97
South Asian *O. rufipogon* (C8)	69	7.97	3.31	6.85	0.10	0.64	0.62	0.86
Southeast Asian *O. rufipogon* (C3)	122	9.48	5.07	7.36	0.10	0.66	0.64	0.85
Australasian *O. rufipogon* (C7)	44	5.38	1.41	5.04	0.07	0.59	0.56	0.88

S, number of samples; A_L_, mean number of alleles per locus; RA_L_, mean number of rare alleles per locus; R_S_, allelic richness; *H*_O_, observed heterozygosity; *H*_E_, gene diversity (unbiased estimate); PIC, polymorphism information content; *F*_IS_, inbreeding coefficient.

Based on allelic richness and gene diversity, C3 (Southeast Asian *O. rufipogon*) is the most diverse among the population groups, followed by C8 (South Asian *O. rufipogon*) (Table 2). The genetic variation in Southeast Asian *O. nivara* clusters C2 and C6 is comparable to that of C7 (Australasian *O. rufipogon*) and greater than those of South Asian *O. nivara* clusters C1 and C5. Next to C4 (*O. meridionalis*), C5 (Nepalese *O. nivara*) shows the least diversity among the population groups. Heterozygosity is greater in the *O. rufipogon* clusters C3, C7, and C8 (0.07–0.1) than in the rest of the population groups (0.01–0.03). Clusters with the highest proportion of rare alleles are C3 (53.5%), C8 (41.6%), and C2 (35.3%) (Table 2).

### Unique and shared alleles

**Figure 7:**
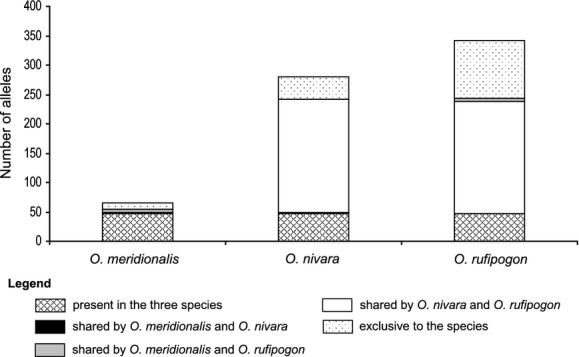
The proportion of private and shared alleles in *Oryza meridionalis, O. nivara,* and *O. rufipogon* in Asia Pacific.

Forty-seven alleles are common to the three species (Fig. 7 and Table S3). *Oryza meridionalis* shares two alleles with *O. nivara* and five alleles with *O. rufipogon*. In stark contrast, *O. nivara* and *O. rufipogon* share 192 alleles making up more than half of the total alleles detected in the annual (68.6%) and perennial (56.1%) taxa. Of the 192 alleles, 14 are exclusively present in Southeast Asian populations (C2, C3, and C6), only one allele is endemic to South Asian populations (C1 and C8), whereas the remaining 177 alleles are not restricted to regionally sympatric populations (Table S3).

Sixteen of the 52 alleles (31%) shared by *O. meridionalis* and *O. rufipogon* are detected in at least one of the five locally sympatric population pairs, whereas the remaining 36 alleles (69%) are shared by nonsympatric populations (Fig. S6). Among the 239 shared alleles of *O. nivara* and *O. rufipogon*, 98 (41%) are found in locally sympatric populations and 141 are found in nonsympatric populations (Fig. S6).

*Oryza rufipogon* has the largest proportion of unique alleles (98 alleles, 28.7%), followed by *O. meridionalis* (11 alleles, 16.9%) and *O. nivara* (39 alleles, 13.9%) (Table S3; Fig. 7). The most highly discriminating markers for *O. meridionalis* are RM124, RM316, and RM413, as they distinguish all accessions of the Australasian species from the rest of Asia-Pacific *Oryza* series *Sativae* populations (Table S3). RM44, RM431, RM118, and RM161 discriminate 12.5–20.8% of *O. meridionalis* populations, whereas RM237 and RM433 distinguish less than 5% of the taxon. Certain alleles of RM154, RM413, RM44, RM433, and RM495 are found exclusively in all geographic populations of *O. rufipogon,* but in limited frequencies ranging from 0.007 to 0.432. RM118 differentiates 47% of Australasian *O. rufipogon,* and at least one allele from each of the 26 loci (RM277, RM455, and RM536 are not included) discriminates a small proportion (allele frequencies ranging from 0.004 to 0.205) of one or two *O. rufipogon* population group/s. No allele is present throughout the distribution range of *O. nivara*. The 39 unique alleles from 20 markers discriminate at least one of the four *O. nivara* population groups in frequencies ranging from 0.007 to 0.304 (Table S3).

### Genetic differentiation

Based on the AMOVA results, genetic variation in Asia-Pacific *Oryza* series *Sativae* (excluding *O. sativa*) resides mainly among accessions (explaining 64% of the total variance) and to a lower degree within accessions (26%) as well as among the three species (10%) (Table 3). Significant and moderate differentiation can be observed between accessions (*Φ*_PT_ = 0.74) and between species (*Φ*_RT_ = 0.1), respectively (both at *P* < 0.001 level).

**Table 3:** Analysis of molecular variance among species, among populations, and within populations of Asia-Pacific *Oryza* series *Sativae* accessions based on 29 SSR markers

Source	Degree of freedom	Sum of squares	Mean sum of squares	Estimated variance	Percentage of variance	*P*-value
Among species	2	1449.896	724.948	4.408	10	0.001
Among populations	100	13,853.252	138.533	27.102	64	0.001
Within populations	382	4205.283	11.009	11.009	26	0.001
Total	484	19,508.431		42.518	100	

The *P*-values are based on 999 permutations.

**Figure 8:**
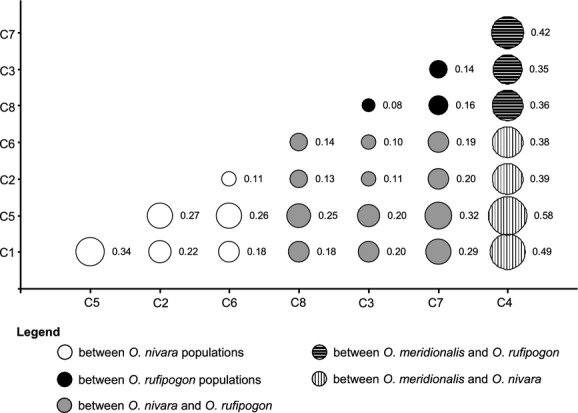
Pairwise *F*_ST_ of the eight population groups of *Oryza* series *Sativae* from Asia Pacific. All values are significant at *P* < 0.001 level based on 999 permutations. C1, Indian and Bangladeshi *O. nivara*; C5, Nepalese *O. nivara*; C2, Cambodian *O. nivara*; C6, non-Cambodian Southeast Asian *O. nivara*; C8, South Asian *O. rufipogon*; C3, Southeast Asian *O. rufipogon*; C7, Australasian *O. rufipogon*; C4, *O. meridionalis*. Circle size is proportional to its corresponding *F*_ST_ value (indicated at its right-hand side).

The population clusters identified by TESS at *K* = 8 display different degrees of differentiation (Fig. 8) with pairwise *F*_ST_ values ranging from 0.08 (between South [C8] and Southeast Asian [C3] *O. rufipogon*) to 0.58 (between *O. meridionalis* [C4] and Nepalese *O. nivara* [C5]). *Oryza meridionalis* is clearly the most distinct population group. The mean pairwise *F*_ST_ value between *O. nivara* clusters (0.23) is greater than between *O. rufipogon* clusters (0.13) and even between clusters of *O. nivara* and *O. rufipogon* (0.19) suggesting deep genetic divisions within the annual species. *Oryza nivara* from Nepal (C5) is clearly differentiated from the rest of the clusters. The Australasian cluster C7 seems the most distinct among the *O. rufipogon* population groups. The pairwise *F*_ST_ values are lower between the Southeast Asian clusters of *O. nivara* (C2 and C6) and *O. rufipogon* (C3) than between the South Asian clusters of the two species.

## Discussion

### Global overlapping and local differentiation

NJ (Fig. 1) and ordination (Fig. 2) methods reveal a lack of clear-cut genetic division between *O. nivara* and *O. rufipogon* across their distribution range, concurring with results of previous molecular studies (Second 1985; Barbier 1989; Iwamoto et al. 1999; Park et al. 2003; Ren et al. 2003; Cai et al. 2004; Zhu and Ge 2005; Zhou et al. 2008; Zheng and Ge 2010; Huang et al. 2012a). None of the markers used in this study can discriminate the majority of either *O. nivara* or *O. rufipogon* accessions from the rest of the series. The relatively large extent of allele sharing between nonsympatric populations from different geographic regions (Table S3 and Fig. S6) renders it more probable that most of the similarities can be traced to common ancestry, although gene flow cannot be ruled out as an explanation for the genetic overlap of the two species (Zhou et al. 2008; Zheng and Ge 2010). However, genetic separation of *O. nivara* and *O. rufipogon* was detected by Bayesian clustering methods at the highest population structure level (*K* = 2), even earlier than the recognition of *O. meridionalis* as a distinct group (at *K* = 3).

Despite the genetic overlap, species separation is apparent at a local scale. *Oryza nivara* and *O. rufipogon* populations from the same locality cluster apart from each other (except N43 and R43) in the NJ tree. Spatially explicit Bayesian clustering differentiated the two species in sympatric population pairs throughout their distribution range (Fig. 5). Kuroda et al. (2007) have reported species separation of *O. nivara* and *O. rufipogon* populations in Vientiane, Laos. Likewise, Singh et al. (2013) observed species divergence in local populations found within the Indo-Gangetic Plains of India. Therefore, contrary to the claim of Zheng and Ge (2010), molecular divergence is not completely absent between the two species and exists locally in sympatric populations indicative of adequately strong barriers to gene flow (e.g., differences in phenology and mating system) operating over smaller spatial units. It is evident from the AMOVA results that gene flow is more restricted between populations than between species (Table 3). Indeed, crossability studies suggest that reproductive barriers between *O. nivara* and *O. rufipogon* as well as between *O. meridionalis* and *O. rufipogon* tend to intensify under sympatric conditions (Banaticla-Hilario et al. 2013).

The failure of molecular data to clearly separate the two taxa led some scientists to treat *O. nivara* as an ecotype of *O. rufipogon* (Ren et al. 2003; Zhu and Ge 2005; Zheng and Ge 2010). However, we might have a different situation at hand. Recently, some authors postulated the acceptance of the “genic view” of speciation (Wu 2001; Lexer and Widmer 2008). In this view, species reproductive barriers are somewhat permeable to gene flow, and speciation can be triggered by expression of relatively few genes that affect differential adaptation and reproductive isolation. These “speciation genes” remain diverged while neutral loci are more freely exchanged between species (Wu 2001; Feder and Nosil 2010; Rieseberg and Blackman 2010; Nosil and Schluter 2011; Southcott and Ostevik 2011). Similar to the case of *O. nivara* and *O. rufipogon,* adaptive divergence in the face of massive allele sharing but resulting in reproductive isolation has been observed in closely related, recently diverged, and geographically overlapping species of *Howea* (Savolainen et al. 2006), *Silene* (Bratteler et al. 2006), *Lupinus* (Drummond and Hamilton 2007), *Helianthus* (Yatabe et al. 2007), and *Pitcairnia* (Palma-Silva et al. 2011), where species discrimination appears to involve only a few loci/genes. Whether *O. nivara* and *O. rufipogon* are “genic” species remains to be seen as their speciation genes still await ascertainment. A good starting point is the work of Grillo et al. (2009), where quantitative trait loci (QTLs) with moderate to large effect on flowering time as well as QTLs with small to moderate effect on floral and panicle traits associated with the mating system of *O. nivara* were identified. The same authors also implicated the role of directional selection in the fixation of majority of the QTL alleles of *O. nivara*. The loci/genes used in earlier studies that confirmed species separation could also hold clues to the identity of their supposed speciation genes. Kuroda et al. (2007) and Singh et al. (2013) used a total of 30 SSR markers that differentiated local populations of *O. nivara* and *O. rufipogon*. Likewise, species divergence was evident in the results of Duan et al. (2007) based on sequences of the chloroplast *trn*L intron and *trn*L-*trn*F spacer, the mitochondrial *nad1* intron 2, and the nuclear internal transcribed spacer, and in those of Xu et al. (2012) based on 6.5 million SNPs.

### Regional divergence

The population groups of *O. nivara* and *O. rufipogon* in South Asia exhibit lower diversity (Table 2) and higher intra- and interspecific differentiation (Fig. 8) than their Southeast Asian counterparts. Moreover, only one allele is exclusively shared by *O. nivara* and *O. rufipogon* in South Asia, whereas 14 alleles are endemic and common between the two species in Southeast Asia. This evidence indicates stronger gene flow barriers in the South Asian region. Such a geographic pattern conforms to the morphological variations reported by Banaticla-Hilario (2012) but contradicts an earlier SSR experiment that reported greater species differentiation in Southeast Asia (Lu et al. 2008).

The optimal four-cluster solution of STRUCTURE recognizes the South Asian and Southeast Asian populations of *O. nivara* as two genetically distinct groups (Figs. 4, 5). *Oryza nivara* is confined to areas with a pronounced dry season and its occurrence has not been reported in the more humid, western part of Myanmar (Vaughan et al. 2008) where the regional boundary of tropical continental Asia lies. This geoclimatic factor probably restricts gene flow between the South and Southeast Asian populations of *O. nivara*. The vicariance displayed by *O. nivara* is also evident from phenotype data (Banaticla-Hilario 2012) and could be a plausible explanation of regional differences in the extent of genetic differentiation between *O. nivara* and *O. rufipogon*.

### Australasian populations are distinct

The results confirmed the genetic disparities of *O. meridionalis* from the rest of the series (Figs. 1, 2, 8) and of the Australasian populations of *O. rufipogon* from the rest of the perennial species (Figs. 4, 5, 8). The latter disagrees with the findings of Huang et al. (2012a) where *O. rufipogon* exhibited two genetic groups (i.e., China-centered Ruf-I and South Asia-centered Ruf-II) displaying a clinal variation pattern. This China–South Asia division cannot be confirmed in the present analysis as only one accession from China was sampled.

Nevertheless, the pattern obtained in this study corresponds to the previously reported morphological (Banaticla-Hilario 2012) and genetic (Waters et al. 2012) divergence of Australasian *O. rufipogon*. Genetic and morphological differentiation between continental and insular populations has been reported in many other plant species (e.g., Howcroft and Davidson 1973; Rivera-Ocasio et al. 2006; Fievet et al. 2007; Fedorenko et al. 2009). Geographic isolation probably restrained gene exchange between these Australasian taxa and the other population groups and may have caused genetic bottlenecks as indicated by the poor genetic diversity of *O. meridionalis* (Table 2). However, a larger number of accessions and multiple individuals from the accessions should be analyzed to confirm this conclusion. *Oryza rufipogon* populations in Australasia have been reported to flourish vegetatively and produce less seeds in their natural habitats (Vaughan et al. 2003, 2008), which could also be a reason for their low genetic diversity.

The geographic separation and low diversity of *O. meridionalis* and Australasian *O. rufipogon* predispose them to inbreeding depression and subsequent genetic deterioration as observed in other island populations (Frankham 1997, 1998; Fedorenko et al. 2009). Therefore, these population groups should be carefully examined and considered in reviewing and designing management practices for their protection and preservation.

### *Oryza nivara* in Nepal: a discrete genetic entity

The genetic distinctiveness of *O. nivara* populations in Nepal is comparable to that of *O. meridionalis* as explicitly shown in the NJ (Fig. 1), ordination (Fig. 2), and *F*_ST_ (Fig. 8) results. However, the Nepalese *O. nivara* group seems distinguishable at lower population structure levels. Bayesian methods detect this group at *K* = 5 following the recognition of the South–Southeast Asia split in *O. nivara* at *K* = 4 (Figs. 4, 5). The uniqueness of *O. meridionalis* is evident also at higher population structure levels (*K* = 3).

Low diversity and genetic isolation from the rest of the species expose the Nepalese *O. nivara* to inbreeding depression and genetic erosion. More in-depth studies are needed not just to confirm the unique genetic identity of these regional populations but also to further establish variation patterns that will aid in formulating in and ex situ conservation strategies.

### Associations with *O. sativa*

The reported consanguinity of *O. rufipogon* with *O. sativa* var. *japonica* and of *O. nivara* with *O. sativa* var. *indica* (Cheng et al. 2003; Yamanaka et al. 2003; Ohtsubo et al. 2004; Xu et al. 2007, 2012) is evident from this study (Figs. 1, 2, 4). At the uppermost hierarchical level of population structure (*K* = 2), the japonica and aromatic varietal groups join up with *O. rufipogon,* whereas the indica and aus groups do so with *O. nivara* (Fig. S1).

Geographic clustering patterns are further displayed by the cultivated varieties at lower structural levels. Starting at *K* = 4, aus consistently groups with Cambodian *O. nivara* (Fig. 4), whereas starting at *K* = 7, indica clusters with *O. nivara* from Thailand. This is analogous to the clustering patterns revealed by 6.5 million SNPs where indica and aus appeared similar to different populations of *O. nivara* (Xu et al. 2012). However, the limited number of cultivated and Chinese wild rice populations analyzed in this study limits the validity of the clustering patterns obtained. Phylogenetic analyses based on ∼8 million SNPs indicated that japonica is genetically closer to *O. rufipogon* from China than to any other *O. rufipogon* populations in Asia (Huang et al. 2012b). Phylogeographic results (Londo et al. 2006) agree with the genetic association of indica with wild rice in Thailand (Fig. 4), but are in discordance with the observed merging of aromatic and japonica with South Asian *O. rufipogon* at *K* = 6 (Fig. 4).

It is worth mentioning that of the six populations morphologically classified as weedy forms (i.e., intermediate between *O. sativa* and either *O. nivara* or *O. rufipogon*) (Banaticla-Hilario 2012), one was detected by STRUCTURE (at *K* = 6) as a genetic admixture of *O. nivara* and *O. rufipogon,* whereas the other populations were included in the *O. nivara*–indica group. Caution should be taken when interpreting SSR diversity patterns as the presence of interaction between cultivated and wild taxa could be masked by the genetic similarities within *Oryza* series *Sativae*. Vaughan et al. (2008) warned that some genebank accessions of the Asian wild rice might have introgressed with cultivated rice, as most of these accessions were collected from disturbed habitats.

## Conclusions

This research imparts a more detailed account of the genetic variation patterns in *O. nivara* and *O. rufipogon*, less so in the geographically restricted *O. meridionalis*. The recognition of local differentiation in the midst of global similarities reconciles the conflicting results of prior studies (Second 1985; Barbier 1989; Aggarwal et al. 1999; Iwamoto et al. 1999; Park et al. 2003; Ren et al. 2003; Cai et al. 2004; Zhu and Ge 2005; Duan et al. 2007; Kuroda et al. 2007; Takahashi et al. 2008; Zhou et al. 2008; Zheng and Ge 2010; Xu et al. 2012; Singh et al. 2013). Furthermore, regional differences in the strength of interspecific gene flow have been detected indicating that the extent of genetic differentiation between *O. nivara* and *O. rufipogon* varies at different geographic scales.

The revealed geographic partitions within species as well as the inferred population groupings within the series can be considered in assessing the genetic representativeness of genebank collections and in selecting plant materials for in and ex situ conservation and research purposes. Especially, the uniqueness and vulnerability to genetic degradation of *O. meridionalis, O. nivara* in Nepal, and *O. rufipogon* in Australasia call for immediate conservation measures. Furthermore, the vast amount of genetic variation detected among populations justifies the maintenance of a large collection of Asian wild rice germplasm.

## Supporting Information

**Figure S1.** Cluster solutions produced by STRUCTURE and TESS from *K* = 2 to *K* = 8. The average membership coefficient of 10 runs from each *K* is shown. The predefined populations are represented as follows: 1, *Oryza sativa* (aromatic); 2, *O. sativa* (japonica); 3, *O. sativa* (indica); 4, *O. sativa* (aus); 5, *O. nivara* from South Asia; 6, *O. nivara* from Southeast Asia; 7, *O. rufipogon* from South Asia; 8, *O. rufipogon* from China; 9, *O. rufipogon* from continental Southeast Asia; 10, *O. rufipogon* from insular Southeast Asia; 11, *O. rufipogon* from Australasia; 12, *O. meridionalis* from Australasia.

**Figure S2.** Membership coefficients of 10 aligned STRUCTURE runs at *K* = 4 and *K* = 6. The predefined populations are represented as follows: 1, *Oryza sativa* (aromatic); 2, *O. sativa* (japonica); 3, *O. sativa* (indica); 4, *O. sativa* (aus); 5, *O. nivara* (from South Asia); 6, *O. nivara* (from Southeast Asia); 7, *O. rufipogon* (from South Asia); 8, *O. rufipogon* (from China); 9, *O. rufipogon* (from continental Southeast Asia); 10, *O. rufipogon* (from insular Southeast Asia); 11, *O. rufipogon* (from Australasia); 12, *O. meridionalis* (from Australasia).

**Figure S3.** (A) Membership coefficients of the 10 aligned TESS runs at *K* = 2 and *K* = 3. The predefined populations are represented as follows: 1, South Asian *Oryza nivara*; 2, Southeast Asian *O. nivara*; 3, South Asian *O. rufipogon*; 4, Chinese *O. rufipogon*; 5, continental Southeast Asian *O. rufipogon*; 6, insular Southeast Asian *O. rufipogon*; 7, Australasian *O. rufipogon*; and 8, *O. meridionalis*. (B) Membership coefficients of the 10 aligned TESS runs at *K* = 4 and *K* = 5. The predefined populations are represented as follows: 1, South Asian *O. nivara*; 2, Southeast Asian *O. nivara*; 3, South Asian *O. rufipogon*; 4, Chinese *O. rufipogon*; 5, continental Southeast Asian *O. rufipogon*; 6, insular Southeast Asian *O. rufipogon*; 7, Australasian *O. rufipogon*; and 8, *O. meridionalis*. (C) Membership coefficients of the 10 aligned TESS runs at *K* = 6 and *K* = 7. The predefined populations are represented as follows: 1, South Asian *O. nivara*; 2, Southeast Asian *O. nivara*; 3, South Asian *O. rufipogon*; 4, Chinese *O. rufipogon*; 5, continental Southeast Asian *O. rufipogon*; 6, insular Southeast Asian *O. rufipogon*; 7, Australasian *O. rufipogon*; and 8, *O. meridionalis*. (D) Membership coefficients of the 10 aligned TESS runs at *K* = 8 and *K* = 9. The predefined populations are represented as follows: 1, South Asian *O. nivara*; 2, Southeast Asian *O. nivara*; 3, South Asian *O. rufipogon*; 4, Chinese *O. rufipogon*; 5, continental Southeast Asian *O. rufipogon*; 6, insular Southeast Asian *O. rufipogon*; 7, Australasian *O. rufipogon*; and 8, *O. meridionalis*. (E) Membership coefficients of the 10 aligned TESS runs at *K* = 10. The predefined populations are represented as follows: 1, South Asian *O. nivara*; 2, Southeast Asian *O. nivara*; 3, South Asian *O. rufipogon*; 4, Chinese *O. rufipogon*; 5, continental Southeast Asian *O. rufipogon*; 6, insular Southeast Asian *O. rufipogon*; 7, Australasian *O. rufipogon*; and 8, *O. meridionalis*.

**Figure S4.** Placement of TESS clusters in the plot of the first two principal coordinate axes (axis 1 on *x* and axis 2 on *y*). (A) at *K* = 4, C1, South Asian *Oryza nivara*; C2, Southeast Asian *O. nivara*; C3, continental Asian and insular Southeast Asian *O. rufipogon*; and C4, *O. meridionalis* and Australasian *O.rufipogon*. (B) at *K* = 7, C1, Indian and Bangladeshi *O. nivara*; C2, Cambodian *O. nivara*; C3, continental Asian and insular Southeast Asian *O. rufipogon*; C4, *O. meridionalis*; C5, Nepalese *O. nivara*; C6, non-Cambodian *O. nivara*; and C7, Australasian *O. rufipogon*. (C) at *K* = 8, C1, Indian and Bangladeshi *O. nivara*; C2, Cambodian *O. nivara*; C3, Southeast Asian *O. rufipogon*; C4, *O. meridionalis*; C5, Nepalese *O. nivara*; C6, non-Cambodian *O. nivara*; C7, Australasian *O. rufipogon*; and C8, South Asian *O. rufipogon*. Admixed populations are those with less than 0.6 membership coefficient.

**Figure S5.** Placement of TESS clusters in the plot of the third and fourth principal coordinate axes (axis 3 on *x* and axis 4 on *y*). (A) at *K* = 4, C1, South Asian *Oryza nivara*; C2, Southeast Asian *O. nivara*; C3, continental Asian and insular Southeast Asian *O. rufipogon*; and C4, *O. meridionalis* and Australasian *O. rufipogon*. (B) at *K* = 7, C1, Indian and Bangladeshi *O. nivara*; C2, Cambodian *O. nivara*; C3, continental Asian and insular Southeast Asian *O. rufipogon*; C4, *O. meridionalis*; C5, Nepalese *O. nivara*; C6, Non-Cambodian *O. nivara*; and C7, Australasian *O. rufipogon*. (C) at *K* = 8, C1, Indian and Bangladeshi *O. nivara*; C2, Cambodian *O. nivara*; C3, Southeast Asian *O. rufipogon*; C4, *O. meridionalis*; C5, Nepalese *O. nivara*; C6, Non-Cambodian *O. nivara*; C7, Australasian *O. rufipogon*; and C8, South Asian *O. rufipogon*. Admixed populations are those with less than 0.6 membership coefficient.

**Figure S6.** Proportion of shared alleles detected in sympatric and nonsympatric population pairs of *Oryza meridionalis* and *O. rufipogon* (MR) and of *O. nivara* and *O. rufipogon* (NR).

**Table S1.** Genetic diversity exhibited by the 125 *Oryza* series *Sativae* populations across 29 microsatellite loci.

**Table S2.** TESS cluster membership of each population group at *K* = 8 (averaged over 10 runs).

Table S3. Allele sizes and frequencies of 29 SSR markers in Asia Pacific *Oryza* series *Sativae*. Alleles that can distinguish all or certain population groups within a species are highlighted. Highly discriminating alleles are boldfaced.
