# Supplementary material for: Local differentiation amidst extensive allele sharing in Oryza nivara and O. rufipogon
Source: Ecol Evol. 2013 Aug 1;3(9):3047–62. doi: 10.1002/ece3.689 (PMC3790550; doi:10.1002/ece3.689)
Supplement: Supplementary file 1 [file ece30003-3047-SD1.doc]

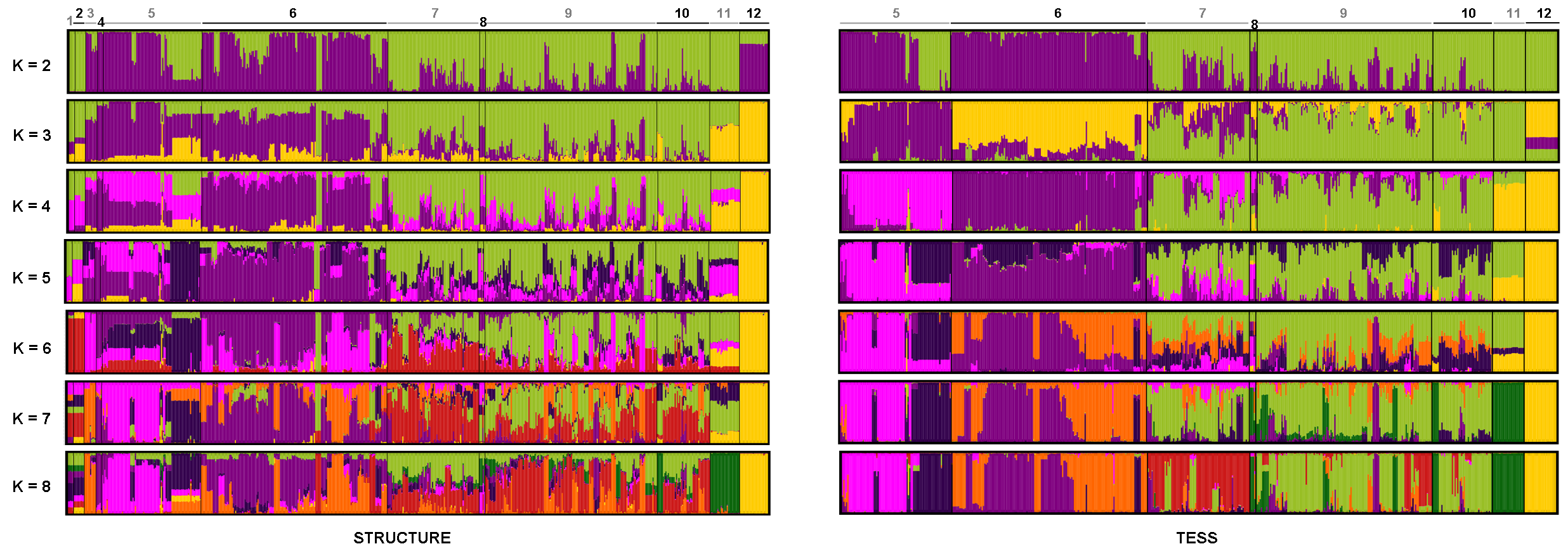


Figure S1. Cluster solutions produced by STRUCTURE and TESS from K = 2 to K = 8. The average membership coefficient of 10 runs from each K are shown. The pre-defined populations are: 1 – *O. sativa* (aromatic); 2 – *O. sativa* (japonica); 3 – *O. sativa* (indica); 4 – *O. sativa* (aus); 5 – *O. nivara* from South Asia; 6 – *O. nivara* from Southeast Asia; 7 – *O. rufipogon* from South Asia; 8 – *O. rufipogon* from China; 9 – *O. rufipogon* from continental Southeast Asia; 10 – *O. rufipogon* from insular Southeast Asia; 11 – *O. rufipogon* from Australasia; 12 – *O. meridionalis* from Australasia.
