# Supplementary material for: Local differentiation amidst extensive allele sharing in Oryza nivara and O. rufipogon
Source: Ecol Evol. 2013 Aug 1;3(9):3047–62. doi: 10.1002/ece3.689 (PMC3790550; doi:10.1002/ece3.689)
Supplement: Supplementary file 2 [file ece30003-3047-SD2.doc]

Figure S2. Membership coefficients of 10 aligned STRUCTURE runs at K = 4 and K = 6. The pre-defined populations are: 1 – *O. sativa* (aromatic); 2 – *O. sativa* (japonica); 3 – *O. sativa* (indica); 4 – *O. sativa* (aus); 5 – *O. nivara* (from South Asia); 6 – *O. nivara* (from Southeast Asia); 7 – *O. rufipogon* (from South Asia); 8 – *O. rufipogon* (from China); 9 – *O. rufipogon* (from continental Southeast Asia); 10 – *O. rufipogon* (from insular Southeast Asia); 11 – *O. rufipogon* (from Australasia); 12 – *O. meridionalis* (from Australasia).
