# Supplementary material for: Local differentiation amidst extensive allele sharing in Oryza nivara and O. rufipogon
Source: Ecol Evol. 2013 Aug 1;3(9):3047–62. doi: 10.1002/ece3.689 (PMC3790550; doi:10.1002/ece3.689)
Supplement: Supplementary file 3 [file ece30003-3047-SD3.doc]

Figure S3A. Membership coefficients of the 10 aligned TESS runs at K = 2 and K = 3. The predefined populations are: 1 – South Asian *O. nivara*; 2 – Southeast Asian *O. nivara*; 3 – South Asian *O. rufipogon*; 4 – Chinese *O. rufipogon*; 5 – continental Southeast Asian *O. rufipogon*; 6 – insular Southeast Asian *O. rufipogon*; 7 – Australasian *O. rufipogon*; and 8 – *O. meridionalis*.

Figure S3B. Membership coefficients of the 10 aligned TESS runs at K = 4 and K = 5. The predefined populations are: 1 – South Asian *O. nivara*; 2 – Southeast Asian *O. nivara*; 3 – South Asian *O. rufipogon*; 4 – Chinese *O. rufipogon*; 5 – continental Southeast Asian *O. rufipogon*; 6 – insular Southeast Asian *O. rufipogon*; 7 – Australasian *O. rufipogon*; and 8 – *O. meridionalis*.

Figure S3C. Membership coefficients of the 10 aligned TESS runs at K = 6 and K = 7. The predefined populations are: 1 – South Asian *O. nivara*; 2 – Southeast Asian *O. nivara*; 3 – South Asian *O. rufipogon*; 4 – Chinese *O. rufipogon*; 5 – continental Southeast Asian *O. rufipogon*; 6 – insular Southeast Asian *O. rufipogon*; 7 – Australasian *O. rufipogon*; and 8 – *O. meridionalis*.

Figure S3D. Membership coefficients of the 10 aligned TESS runs at K = 8 and K = 9. The predefined populations are: 1 – South Asian *O. nivara*; 2 – Southeast Asian *O. nivara*; 3 – South Asian *O. rufipogon*; 4 – Chinese *O. rufipogon*; 5 – continental Southeast Asian *O. rufipogon*; 6 – insular Southeast Asian *O. rufipogon*; 7 – Australasian *O. rufipogon*; and 8 – *O. meridionalis*.

Figure S3E. Membership coefficients of the 10 aligned TESS runs at K = 10. The predefined populations are: 1 – South Asian *O. nivara*; 2 – Southeast Asian *O. nivara*; 3 – South Asian *O. rufipogon*; 4 – Chinese *O. rufipogon*; 5 – continental Southeast Asian *O. rufipogon*; 6 – insular Southeast Asian *O. rufipogon*; 7 – Australasian *O. rufipogon*; and 8 – *O. meridionalis*.
