# Supplementary material for: Local differentiation amidst extensive allele sharing in Oryza nivara and O. rufipogon
Source: Ecol Evol. 2013 Aug 1;3(9):3047–62. doi: 10.1002/ece3.689 (PMC3790550; doi:10.1002/ece3.689)
Supplement: Supplementary file 4 [file ece30003-3047-SD4.doc]

Figure S4. Placement of TESS clusters in the plot of the first two principal coordinate axes (axis 1 on x and axis 2 on y). **A)** at K = 4, C1 - South Asian *O. nivara*; C2 - Southeast Asian *O. nivara*; C3 – continental Asian and insular Southeast Asian *O. rufipogon*; and C4 - *O. meridionalis* and Australasian *O.rufipogon*. **B)** at K = 7, C1 - Indian and Bangladeshi *O. nivara*; C2 - Cambodian *O. nivara*; C3 – continental Asian and insular Southeast Asian *O. rufipogon*; C4 - *O. meridionalis*; C5 - Nepalese *O. nivara*; C6 - non-Cambodian *O. nivara*; and C7 - Australasian *O. rufipogon*. **C)** at K = 8, C1 - Indian and Bangladeshi *O. nivara*; C2 - Cambodian *O. nivara*; C3 – Southeast Asian *O. rufipogon*; C4 - *O. meridionalis*; C5 - Nepalese *O. nivara*; C6 - non-Cambodian *O. nivara*; C7 - Australasian *O. rufipogon*; and C8 – South Asian *O. rufipogon.* Admixed populations are those with less than 0.6 membership coefficient.
