# Supplementary material for: Local differentiation amidst extensive allele sharing in Oryza nivara and O. rufipogon
Source: Ecol Evol. 2013 Aug 1;3(9):3047–62. doi: 10.1002/ece3.689 (PMC3790550; doi:10.1002/ece3.689)
Supplement: Supplementary file 6 [file ece30003-3047-SD6.doc]

Figure S6. Proportion of shared alleles detected in sympatric and non-sympatric population pairs of *O. meridionalis* and *O. rufipogon* (MR) and of *O. nivara* and *O. rufipogon* (NR).
