# Supplementary material for: Local differentiation amidst extensive allele sharing in Oryza nivara and O. rufipogon
Source: Ecol Evol. 2013 Aug 1;3(9):3047–62. doi: 10.1002/ece3.689 (PMC3790550; doi:10.1002/ece3.689)
Supplement: Supplementary file 7 [file ece30003-3047-SD7.doc]

Table S1. Genetic diversity exhibited by the 125 *Oryza* series *Sativae* populations across 29 microsatellite loci.

| Code | IRGC number | Species | Geographic origin | Coordinates | | Sample size | Number of alleles | Allelic richness | Hetero-zygosity | Gene diversity |
| --- | --- | --- | --- | --- | --- | --- | --- | --- | --- | --- |
|  |  |  |  | Latitude | Longitude |  |  |  |  |  |
| N1 | 105882 | *O. nivara* | Bangladesh | 23.0166 | 89.4666 | 5 | 1.55 | 1.22 | 0.02 | 0.16 |
| N2 | 103830 | *O. nivara* | Bangladesh | 24.3333 | 88.9000 | 5 | 1.07 | 1.03 | 0.00 | 0.02 |
| N18 | 80549 | *O. nivara* | India | 19.6667 | 81.6833 | 5 | 1.07 | 1.03 | 0.01 | 0.02 |
| N19 | 80560 | *O. nivara* | India | 19.7500 | 81.3333 | 4 | 1.24 | 1.09 | 0.04 | 0.07 |
| N20 | 80601 | *O. nivara* | India | 20.0000 | 81.5000 | 5 | 1.21 | 1.08 | 0.02 | 0.06 |
| N21 | 80677 | *O. nivara* | India | 21.1333 | 81.3333 | 5 | 1.69 | 1.26 | 0.03 | 0.19 |
| N22 | 106101 | *O. nivara* | India | 21.7500 | 87.5000 | 5 | 1.34 | 1.15 | 0.03 | 0.11 |
| N23 | 80645 | *O. nivara* | India | 22.0333 | 81.9167 | 5 | 1.34 | 1.13 | 0.01 | 0.09 |
| N24 | 106051 | *O. nivara* | India | 22.0833 | 86.4167 | 5 | 1.21 | 1.08 | 0.01 | 0.06 |
| N25 | 106054 | *O. nivara* | India | 22.1667 | 86.0000 | 5 | 1.28 | 1.10 | 0.01 | 0.07 |
| N26^a^ | 81837 | *O. nivara* | India | 25.5000 | 84.3333 | 5 | 2.10 | 1.20 | 0.03 | 0.31 |
| N26A | 81837 | *O. nivara* | India | 25.5000 | 84.3333 | 3 | 1.52 | 1.28 | 0.02 | 0.15 |
| N26B | 81837 | *O. nivara* | India | 25.5000 | 84.3333 | 2 | 1.21 | 1.13 | 0.03 | 0.05 |
| N32 | 93192 | *O. nivara* | Nepal | 27.5119 | 83.2428 | 5 | 1.31 | 1.12 | 0.03 | 0.09 |
| N33 | 93185 | *O. nivara* | Nepal | 28.0200 | 81.6033 | 5 | 1.24 | 1.09 | 0.04 | 0.07 |
| N34 | 93191 | *O. nivara* | Nepal | 28.0211 | 81.6011 | 5 | 1.38 | 1.13 | 0.02 | 0.10 |
| N35 | 93184 | *O. nivara* | Nepal | 28.0283 | 81.6322 | 5 | 1.62 | 1.22 | 0.03 | 0.16 |
| N36 | 93195 | *O. nivara* | Nepal | 28.1008 | 82.2683 | 5 | 1.45 | 1.18 | 0.03 | 0.13 |
| N37 | 103422 | *O. nivara* | Sri Lanka | 8.9167 | 81.0000 | 5 | 1.21 | 1.08 | 0.00 | 0.06 |
| N3^bc^ | 93129 | *O. nivara* | Cambodia | 10.6333 | 103.7833 | 3 | 1.34 | 1.17 | 0.05 | 0.10 |
| N4^c^ | 105719 | *O. nivara* | Cambodia | 11.4167 | 104.9667 | 5 | 1.31 | 1.09 | 0.01 | 0.07 |
| N5^c^ | 89216 | *O. nivara* | Cambodia | 11.4467 | 104.4889 | 5 | 1.59 | 1.25 | 0.02 | 0.18 |
| N6 | 89187 | *O. nivara* | Cambodia | 11.4969 | 104.8183 | 5 | 1.59 | 1.21 | 0.04 | 0.15 |
| N7 | 89172 | *O. nivara* | Cambodia | 11.5094 | 104.8189 | 5 | 1.24 | 1.08 | 0.01 | 0.06 |
| N8 | 89185 | *O. nivara* | Cambodia | 11.5297 | 104.8342 | 5 | 1.55 | 1.24 | 0.01 | 0.17 |
| N9 | 89212 | *O. nivara* | Cambodia | 11.5317 | 104.8283 | 5 | 1.38 | 1.14 | 0.02 | 0.10 |
| N10 | 106320 | *O. nivara* | Cambodia | 11.8333 | 104.7933 | 5 | 1.52 | 1.19 | 0.01 | 0.14 |

Table S1. continued

| Code | IRGC number | Species | Geographic origin | Coordinates | | Sample size | Number of alleles | Allelic richness | Hetero-zygosity | Gene diversity |
| --- | --- | --- | --- | --- | --- | --- | --- | --- | --- | --- |
|  |  |  |  | Latitude | Longitude |  |  |  |  |  |
| N11^c^ | 88939 | *O. nivara* | Cambodia | 12.5333 | 106.6166 | 5 | 1.38 | 1.15 | 0.04 | 0.11 |
| N12 | 92886 | *O. nivara* | Cambodia | 12.6166 | 103.9833 | 5 | 1.17 | 1.07 | 0.02 | 0.05 |
| N13 | 92677 | *O. nivara* | Cambodia | 12.6500 | 104.9166 | 5 | 1.52 | 1.21 | 0.03 | 0.16 |
| N14 | 92823 | *O. nivara* | Cambodia | 13.0500 | 104.5166 | 5 | 1.83 | 1.35 | 0.03 | 0.25 |
| N15 | 106339 | *O. nivara* | Cambodia | 13.3833 | 103.8333 | 5 | 1.48 | 1.18 | 0.02 | 0.13 |
| N16 | 92943 | *O. nivara* | Cambodia | 14.1667 | 103.2667 | 5 | 1.34 | 1.14 | 0.02 | 0.10 |
| N27 | 86699 | *O. nivara* | Laos | 15.4100 | 106.7000 | 5 | 1.31 | 1.12 | 0.01 | 0.08 |
| N28 | 106148 | *O. nivara* | Laos | 17.9167 | 102.7500 | 5 | 1.24 | 1.10 | 0.01 | 0.08 |
| N29 | 106151 | *O. nivara* | Laos | 18.2000 | 102.7500 | 4 | 1.24 | 1.10 | 0.02 | 0.07 |
| N31^c^ | 106347 | *O. nivara* | Myanmar | 17.2500 | 96.6667 | 5 | 1.45 | 1.17 | 0.01 | 0.13 |
| N38 | 105803 | *O. nivara* | Thailand | 17.0833 | 104.0833 | 5 | 1.48 | 1.21 | 0.01 | 0.15 |
| N39 | 104724 | *O. nivara* | Thailand | 14.4667 | 100.1166 | 5 | 1.21 | 1.07 | 0.01 | 0.05 |
| N40 | 105765 | *O. nivara* | Thailand | 14.3333 | 102.9167 | 5 | 1.59 | 1.20 | 0.02 | 0.14 |
| N41 | 105755 | *O. nivara* | Thailand | 14.5000 | 102.0833 | 4 | 1.03 | 1.02 | 0.00 | 0.01 |
| N42 | 104743 | *O. nivara* | Thailand | 14.9666 | 102.1166 | 5 | 1.17 | 1.07 | 0.00 | 0.05 |
| N43^c^ | 104756 | *O. nivara* | Thailand | 15.1000 | 104.3333 | 5 | 1.14 | 1.06 | 0.01 | 0.04 |
| N44 | 105859 | *O. nivara* | Thailand | 14.6667 | 102.3333 | 5 | 1.45 | 1.17 | 0.02 | 0.13 |
| N45 | 104473 | *O. nivara* | Thailand | 16.8333 | 100.2500 | 2 | 1.41 | 1.26 | 0.05 | 0.11 |
| N46 | 105801 | *O. nivara* | Thailand | 17.0000 | 104.0833 | 5 | 1.34 | 1.10 | 0.02 | 0.08 |
| N47 | 105809 | *O. nivara* | Thailand | 17.2500 | 103.7500 | 5 | 1.69 | 1.25 | 0.03 | 0.18 |
| N48 | 105825 | *O. nivara* | Thailand | 17.6667 | 102.9167 | 5 | 1.07 | 1.03 | 0.00 | 0.02 |
| N49 | 105828 | *O. nivara* | Thailand | 17.8450 | 102.5841 | 5 | 1.59 | 1.21 | 0.03 | 0.15 |
| N50 | 104736 | *O. nivara* | Thailand | 18.1500 | 100.1333 | 5 | 1.21 | 1.07 | 0.02 | 0.05 |
| N51 | 86496 | *O. nivara* | Vietnam | 12.5681 | 107.7556 | 5 | 1.10 | 1.04 | 0.03 | 0.03 |
| N52 | 86493 | *O. nivara* | Vietnam | 13.6178 | 108.1171 | 5 | 1.21 | 1.07 | 0.02 | 0.05 |
| R1 | 105881 | *O. rufipogon* | Bangladesh | 23.0666 | 89.3500 | 4 | 1.45 | 1.19 | 0.08 | 0.13 |
| R2 | 103827 | *O. rufipogon* | Bangladesh | 24.1500 | 89.0500 | 5 | 1.83 | 1.25 | 0.12 | 0.19 |
| R18 | 80550 | *O. rufipogon* | India | 19.6667 | 81.7667 | 4 | 1.55 | 1.22 | 0.13 | 0.16 |

Table S1. continued

| Code | IRGC number | Species | Geographic origin | Coordinates | | Sample size | Number of alleles | Allelic richness | Hetero-zygosity | Gene diversity |
| --- | --- | --- | --- | --- | --- | --- | --- | --- | --- | --- |
|  |  |  |  | Latitude | Longitude |  |  |  |  |  |
| R19 | 80562 | *O. rufipogon* | India | 19.7500 | 81.3167 | 5 | 1.59 | 1.22 | 0.07 | 0.16 |
| R20 | 80600 | *O. rufipogon* | India | 20.0000 | 81.5000 | 4 | 1.38 | 1.14 | 0.06 | 0.10 |
| R21 | 80680 | *O. rufipogon* | India | 21.1333 | 81.5667 | 5 | 1.48 | 1.18 | 0.07 | 0.13 |
| R22 | 82983 | *O. rufipogon* | India | 21.7500 | 87.5000 | 5 | 1.79 | 1.28 | 0.11 | 0.21 |
| R23^c^ | 80643 | *O. rufipogon* | India | 22.0333 | 82.8167 | 5 | 1.76 | 1.28 | 0.23 | 0.22 |
| R24 | 82982 | *O. rufipogon* | India | 22.0833 | 86.3667 | 4 | 1.69 | 1.26 | 0.12 | 0.18 |
| R25 | 106055 | *O. rufipogon* | India | 22.1667 | 85.9167 | 5 | 1.97 | 1.32 | 0.12 | 0.24 |
| R26 | 81881 | *O. rufipogon* | India | 25.5000 | 84.1333 | 3 | 1.62 | 1.28 | 0.07 | 0.17 |
| R32^c^ | 93221 | *O. rufipogon* | Nepal | 27.5119 | 83.2439 | 4 | 1.97 | 1.37 | 0.14 | 0.26 |
| R33 | 93218 | *O. rufipogon* | Nepal | 28.0200 | 81.6033 | 5 | 1.59 | 1.24 | 0.03 | 0.18 |
| R34 | 93220 | *O. rufipogon* | Nepal | 28.0211 | 81.6011 | 5 | 1.76 | 1.31 | 0.08 | 0.23 |
| R35 | 93210 | *O. rufipogon* | Nepal | 28.0283 | 81.6322 | 5 | 1.48 | 1.17 | 0.10 | 0.13 |
| R36 | 93216 | *O. rufipogon* | Nepal | 28.1008 | 82.2683 | 5 | 1.45 | 1.18 | 0.11 | 0.14 |
| R37^c^ | 103423 | *O. rufipogon* | Sri Lanka | 8.5000 | 81.1667 | 5 | 1.34 | 1.15 | 0.03 | 0.11 |
| R17^c^ | 103823 | *O. rufipogon* | China | 23.2333 | 114.0333 | 5 | 1.72 | 1.24 | 0.02 | 0.17 |
| R3 | 93085 | *O. rufipogon* | Cambodia | 10.6333 | 103.7833 | 5 | 2.14 | 1.39 | 0.10 | 0.29 |
| R4 | 105720 | *O. rufipogon* | Cambodia | 11.4167 | 104.9833 | 5 | 1.59 | 1.23 | 0.03 | 0.17 |
| R5^a^ | 89228 | *O. rufipogon* | Cambodia | 11.4467 | 104.4889 | 5 | 2.03 | 1.30 | 0.16 | 0.27 |
| R5A^c^ | 89228 | *O. rufipogon* | Cambodia | 11.4467 | 104.4889 | 3 | 1.62 | 1.28 | 0.15 | 0.17 |
| R5B | 89228 | *O. rufipogon* | Cambodia | 11.4467 | 104.4889 | 2 | 1.59 | 1.32 | 0.17 | 0.15 |
| R6 | 89230 | *O. rufipogon* | Cambodia | 11.4969 | 104.8183 | 5 | 1.83 | 1.31 | 0.09 | 0.23 |
| R7 | 89223 | *O. rufipogon* | Cambodia | 11.5094 | 104.8189 | 5 | 1.62 | 1.26 | 0.06 | 0.19 |
| R8 | 89227 | *O. rufipogon* | Cambodia | 11.5297 | 104.8342 | 2 | 1.69 | 1.37 | 0.17 | 0.17 |
| R9 | 89232 | *O. rufipogon* | Cambodia | 11.5317 | 104.8283 | 5 | 2.34 | 1.40 | 0.16 | 0.30 |
| R10^c^ | 106321 | *O. rufipogon* | Cambodia | 11.8333 | 104.7833 | 4 | 1.31 | 1.12 | 0.08 | 0.09 |
| R11 | 89007 | *O. rufipogon* | Cambodia | 12.5333 | 106.6166 | 5 | 1.69 | 1.25 | 0.08 | 0.19 |
| R12 | 110408 | *O. rufipogon* | Cambodia | 12.6166 | 103.9833 | 5 | 1.97 | 1.35 | 0.08 | 0.26 |

Table S1. continued

| Code | IRGC number | Species | Geographic origin | Coordinates | | Sample size | Number of alleles | Allelic richness | Hetero-zygosity | Gene diversity |
| --- | --- | --- | --- | --- | --- | --- | --- | --- | --- | --- |
|  |  |  |  | Latitude | Longitude |  |  |  |  |  |
| R13 | 99538 | *O. rufipogon* | Cambodia | 12.6500 | 104.9166 | 5 | 2.03 | 1.33 | 0.16 | 0.25 |
| R14 | 93063 | *O. rufipogon* | Cambodia | 13.0500 | 104.5166 | 5 | 2.03 | 1.33 | 0.12 | 0.25 |
| R15^c^ | 106335 | *O. rufipogon* | Cambodia | 13.3833 | 103.8500 | 5 | 1.55 | 1.24 | 0.03 | 0.18 |
| R16 | 110409 | *O. rufipogon* | Cambodia | 14.1667 | 103.2667 | 5 | 1.48 | 1.21 | 0.06 | 0.15 |
| R28 | 106149 | *O. rufipogon* | Laos | 17.9167 | 102.7500 | 5 | 1.79 | 1.28 | 0.08 | 0.21 |
| R29^a^ | 106152 | *O. rufipogon* | Laos | 18.2000 | 102.7500 | 4 | 1.93 | 1.22 | 0.09 | 0.24 |
| R29A^c^ | 106152 | *O. rufipogon* | Laos | 18.2000 | 102.7500 | 2 | 1.45 | 1.27 | 0.12 | 0.11 |
| R29B | 106152 | *O. rufipogon* | Laos | 18.2000 | 102.7500 | 2 | 1.31 | 1.18 | 0.07 | 0.08 |
| R30 | 106357 | *O. rufipogon* | Myanmar | 16.6667 | 96.5000 | 5 | 1.10 | 1.04 | 0.03 | 0.03 |
| R31 | 106346 | *O. rufipogon* | Myanmar | 17.2500 | 96.6333 | 5 | 1.72 | 1.29 | 0.08 | 0.21 |
| R38 | 105804 | *O. rufipogon* | Thailand | 14.4667 | 100.1166 | 4 | 1.28 | 1.12 | 0.04 | 0.08 |
| R39 | 104713 | *O. rufipogon* | Thailand | 14.5000 | 102.0833 | 4 | 1.69 | 1.27 | 0.05 | 0.18 |
| R40 | 105766 | *O. rufipogon* | Thailand | 14.5000 | 102.9167 | 5 | 1.55 | 1.17 | 0.08 | 0.13 |
| R41 | 105758 | *O. rufipogon* | Thailand | 14.6667 | 102.3333 | 5 | 1.69 | 1.25 | 0.10 | 0.19 |
| R42 | 104742 | *O. rufipogon* | Thailand | 14.9666 | 102.1166 | 5 | 1.69 | 1.25 | 0.11 | 0.18 |
| R43^c^ | 104757 | *O. rufipogon* | Thailand | 15.1000 | 104.3333 | 4 | 1.17 | 1.08 | 0.00 | 0.05 |
| R44 | 105860 | *O. rufipogon* | Thailand | 16.8333 | 100.2500 | 4 | 1.38 | 1.17 | 0.03 | 0.11 |
| R46 | 105800 | *O. rufipogon* | Thailand | 17.0833 | 104.0833 | 5 | 1.72 | 1.27 | 0.06 | 0.20 |
| R47 | 82979 | *O. rufipogon* | Thailand | 17.1667 | 103.7500 | 5 | 1.97 | 1.30 | 0.17 | 0.23 |
| R48 | 105823 | *O. rufipogon* | Thailand | 17.6667 | 102.9167 | 2 | 1.24 | 1.16 | 0.05 | 0.07 |
| R49^c^ | 105829 | *O. rufipogon* | Thailand | 17.8450 | 102.5841 | 4 | 1.41 | 1.17 | 0.09 | 0.12 |
| R50^c^ | 104737 | *O. rufipogon* | Thailand | 18.1500 | 100.1333 | 5 | 1.28 | 1.11 | 0.01 | 0.08 |
| R51 | 86512 | *O. rufipogon* | Vietnam | 12.5681 | 107.7556 | 4 | 1.79 | 1.29 | 0.18 | 0.21 |
| R52 | 86506 | *O. rufipogon* | Vietnam | 13.8333 | 107.8667 | 5 | 1.72 | 1.26 | 0.06 | 0.19 |
| R53 | 80774 | *O. rufipogon* | Philippines | 7.8803 | 125.0061 | 5 | 1.48 | 1.18 | 0.07 | 0.14 |
| R54 | 81976 | *O. rufipogon* | Indonesia | -6.5569 | 106.7617 | 5 | 1.59 | 1.24 | 0.06 | 0.17 |
| R55 | 81977 | *O. rufipogon* | Indonesia | -6.1081 | 106.7097 | 5 | 1.55 | 1.20 | 0.11 | 0.15 |

Table S1. continued

| Code | IRGC number | Species | Geographic origin | Coordinates | | Sample size | Number of alleles | Allelic richness | Hetero-zygosity | Gene diversity |
| --- | --- | --- | --- | --- | --- | --- | --- | --- | --- | --- |
|  |  |  |  | Latitude | Longitude |  |  |  |  |  |
| R56 | 81978 | *O. rufipogon* | Indonesia | -6.3333 | 106.3000 | 5 | 2.21 | 1.42 | 0.18 | 0.31 |
| R58 | 105567 | *O. rufipogon* | Indonesia | -0.7500 | 117.2667 | 5 | 2.00 | 1.39 | 0.16 | 0.29 |
| R59 | 105952 | *O. rufipogon* | Indonesia | -6.4167 | 107.0000 | 5 | 1.66 | 1.17 | 0.10 | 0.13 |
| R60 | 105958 | *O. rufipogon* | Indonesia | -6.0631 | 106.1189 | 5 | 1.90 | 1.37 | 0.18 | 0.28 |
| R61 | 106452 | *O. rufipogon* | Indonesia | -3.2500 | 104.6667 | 5 | 1.59 | 1.24 | 0.14 | 0.19 |
| R62 | 106453 | *O. rufipogon* | Indonesia | -3.0833 | 104.5000 | 5 | 1.41 | 1.14 | 0.02 | 0.10 |
| R63 | 86542 | *O. rufipogon* | Australia | -12.5333 | 131.7167 | 5 | 1.55 | 1.22 | 0.17 | 0.17 |
| R64 | 93274 | *O. rufipogon* | Indonesia | -8.2930 | 140.4089 | 5 | 1.21 | 1.06 | 0.01 | 0.04 |
| R65^cd^ | 105283 | *O. rufipogon* | Australia | -12.5833 | 131.3333 | 5 | 1.38 | 1.17 | 0.06 | 0.13 |
| R66 | 105293 | *O. rufipogon* | Australia | -15.6667 | 145.2500 | 5 | 1.10 | 1.04 | 0.02 | 0.03 |
| R67 | 105303 | *O. rufipogon* | Australia | -13.5000 | 141.7500 | 5 | 1.62 | 1.24 | 0.14 | 0.19 |
| M1 | 86539 | *O. meridionalis* | Australia | -12.5333 | 131.7167 | 5 | 1.10 | 1.04 | 0.00 | 0.03 |
| M2 | 93260 | *O. meridionalis* | Indonesia | -8.2835 | 140.4592 | 5 | 1.00 | 1.00 | 0.00 | 0.00 |
| M3 | 105282 | *O. meridionalis* | Australia | -12.5833 | 131.3333 | 4 | 1.10 | 1.04 | 0.01 | 0.03 |
| M4 | 105294 | *O. meridionalis* | Australia | -15.6667 | 145.2500 | 5 | 1.41 | 1.21 | 0.00 | 0.15 |
| M5 | 105302 | *O. meridionalis* | Australia | -13.5000 | 141.7500 | 5 | 1.34 | 1.11 | 0.02 | 0.08 |
| AR1 | 12880 | *O. sativa* | Iran |  |  | 5 | 1.21 | 1.08 | 0.00 | 0.06 |
| J1 | 328 | *O. sativa* | Philippines |  |  | 5 | 1.38 | 1.03 | 0.01 | 0.02 |
| J2 | 12731 | *O. sativa* | Japan |  |  | 5 | 1.07 | 1.12 | 0.00 | 0.09 |
| IN1 | 66970 | *O. sativa* | Philippines |  |  | 5 | 1.24 | 1.03 | 0.03 | 0.02 |
| IN2 | 108921 | *O. sativa* | India |  |  | 5 | 1.07 | 1.09 | 0.00 | 0.07 |
| AU1 | 32561 | *O. sativa* | India |  |  | 4 | 1.28 | 1.14 | 0.05 | 0.10 |

^a^ Composed of two plant types; partitioned into two subpopulations in the succeeding analyses

^b^ Currently labeled as *O. rufipogon* in the International Rice Genebank Collection Information System (IRGCIS), tentatively re-classified by the author as *O. nivara* based on seed morphology

^c^ Classified as intermediate form in previous morphological analysis

^d^ Currently labeled as *O. meridionalis* in IRGCIS, tentatively re-classified by the author as *O. rufipogon* based on seed morphology
