# Supplementary material for: Local differentiation amidst extensive allele sharing in Oryza nivara and O. rufipogon
Source: Ecol Evol. 2013 Aug 1;3(9):3047–62. doi: 10.1002/ece3.689 (PMC3790550; doi:10.1002/ece3.689)
Supplement: Supplementary file 8 [file ece30003-3047-SD8.doc]

Table S2. TESS cluster membership of each population group at K = 8 (averaged over 10 runs).

| Population code | Partial membership to each cluster | | | | | | | |
| --- | --- | --- | --- | --- | --- | --- | --- | --- |
|  | C1 | C2 | C3 | C4 | C5 | C6 | C7 | C8 |
| N1 | 0.5888 | 0.1442 | 0.0033 | 0.0004 | 0.0005 | 0.2592 | 0.0004 | 0.0032 |
| N2 | 0.9905 | 0.0044 | 0.0004 | 0.0002 | 0.0003 | 0.0027 | 0.0001 | 0.0014 |
| N18 | 0.9967 | 0.0003 | 0.0009 | 0.0000 | 0.0005 | 0.0005 | 0.0001 | 0.0011 |
| N19 | 0.9910 | 0.0005 | 0.0028 | 0.0001 | 0.0018 | 0.0017 | 0.0001 | 0.0021 |
| N20 | 0.9965 | 0.0005 | 0.0007 | 0.0000 | 0.0006 | 0.0004 | 0.0001 | 0.0012 |
| N21 | 0.4343 | 0.0017 | 0.0029 | 0.0001 | 0.5394 | 0.0020 | 0.0008 | 0.0188 |
| N22 | 0.9939 | 0.0008 | 0.0010 | 0.0001 | 0.0010 | 0.0012 | 0.0001 | 0.0021 |
| N23 | 0.9902 | 0.0011 | 0.0016 | 0.0002 | 0.0011 | 0.0015 | 0.0001 | 0.0041 |
| N24 | 0.9941 | 0.0005 | 0.0007 | 0.0001 | 0.0010 | 0.0008 | 0.0001 | 0.0027 |
| N25 | 0.9834 | 0.0008 | 0.0061 | 0.0001 | 0.0035 | 0.0015 | 0.0010 | 0.0036 |
| N26A | 0.0111 | 0.0038 | 0.0188 | 0.1729 | 0.4794 | 0.0027 | 0.1624 | 0.1489 |
| N26B | 0.9534 | 0.0169 | 0.0053 | 0.0002 | 0.0025 | 0.0051 | 0.0083 | 0.0085 |
| N37 | 0.2048 | 0.0016 | 0.0936 | 0.0002 | 0.6968 | 0.0005 | 0.0001 | 0.0024 |
| N32 | 0.0004 | 0.0003 | 0.0004 | 0.0000 | 0.9974 | 0.0004 | 0.0001 | 0.0009 |
| N33 | 0.0003 | 0.0004 | 0.0004 | 0.0000 | 0.9974 | 0.0004 | 0.0001 | 0.0010 |
| N34 | 0.0003 | 0.0003 | 0.0004 | 0.0001 | 0.9973 | 0.0004 | 0.0001 | 0.0009 |
| N35 | 0.0022 | 0.0024 | 0.0383 | 0.0001 | 0.9453 | 0.0082 | 0.0015 | 0.0020 |
| N36 | 0.0342 | 0.0013 | 0.0010 | 0.0000 | 0.9565 | 0.0055 | 0.0001 | 0.0013 |
| N51 | 0.0004 | 0.0998 | 0.0020 | 0.0003 | 0.0002 | 0.8947 | 0.0015 | 0.0011 |
| N52 | 0.0007 | 0.0666 | 0.0020 | 0.0002 | 0.0005 | 0.9284 | 0.0003 | 0.0014 |
| N27 | 0.0026 | 0.9843 | 0.0034 | 0.0002 | 0.0003 | 0.0041 | 0.0017 | 0.0034 |
| N28 | 0.1292 | 0.1456 | 0.0023 | 0.0003 | 0.0003 | 0.7206 | 0.0005 | 0.0012 |
| N29 | 0.0027 | 0.0747 | 0.0013 | 0.0002 | 0.0010 | 0.9134 | 0.0010 | 0.0058 |
| N3 | 0.0042 | 0.8200 | 0.0119 | 0.0005 | 0.0053 | 0.1548 | 0.0008 | 0.0025 |
| N4 | 0.0005 | 0.9794 | 0.0024 | 0.0013 | 0.0007 | 0.0142 | 0.0007 | 0.0007 |
| N5 | 0.0018 | 0.9933 | 0.0016 | 0.0001 | 0.0007 | 0.0013 | 0.0004 | 0.0008 |
| N6 | 0.0005 | 0.9730 | 0.0202 | 0.0002 | 0.0023 | 0.0018 | 0.0008 | 0.0011 |
| N7 | 0.0005 | 0.9958 | 0.0013 | 0.0001 | 0.0006 | 0.0009 | 0.0003 | 0.0005 |
| N8 | 0.0009 | 0.9536 | 0.0038 | 0.0002 | 0.0027 | 0.0250 | 0.0008 | 0.0130 |
| N9 | 0.0020 | 0.9881 | 0.0023 | 0.0002 | 0.0035 | 0.0014 | 0.0010 | 0.0013 |
| N10 | 0.0011 | 0.9662 | 0.0013 | 0.0002 | 0.0002 | 0.0277 | 0.0007 | 0.0026 |
| N11 | 0.0010 | 0.0047 | 0.0022 | 0.0051 | 0.0023 | 0.9814 | 0.0005 | 0.0028 |
| N12 | 0.0004 | 0.8945 | 0.0017 | 0.0003 | 0.0024 | 0.0997 | 0.0006 | 0.0004 |
| N13 | 0.0016 | 0.9848 | 0.0033 | 0.0009 | 0.0004 | 0.0070 | 0.0004 | 0.0017 |

Table S2. continued

| Population code | Partial membership to each cluster | | | | | | | |
| --- | --- | --- | --- | --- | --- | --- | --- | --- |
|  | C1 | C2 | C3 | C4 | C5 | C6 | C7 | C8 |
| N14 | 0.0007 | 0.9904 | 0.0011 | 0.0006 | 0.0011 | 0.0049 | 0.0004 | 0.0007 |
| N15 | 0.0018 | 0.7955 | 0.0031 | 0.0007 | 0.0016 | 0.1962 | 0.0004 | 0.0008 |
| N16 | 0.0075 | 0.5837 | 0.0069 | 0.0013 | 0.0007 | 0.3963 | 0.0013 | 0.0025 |
| N38 | 0.0284 | 0.0162 | 0.0007 | 0.0032 | 0.0007 | 0.9463 | 0.0008 | 0.0036 |
| N40 | 0.0023 | 0.0072 | 0.0028 | 0.0006 | 0.0939 | 0.8922 | 0.0004 | 0.0006 |
| N41 | 0.0014 | 0.0033 | 0.0020 | 0.0002 | 0.0008 | 0.9900 | 0.0012 | 0.0012 |
| N42 | 0.0007 | 0.0009 | 0.0014 | 0.0001 | 0.0007 | 0.9944 | 0.0009 | 0.0009 |
| N43 | 0.0004 | 0.0009 | 0.0013 | 0.0007 | 0.0005 | 0.9951 | 0.0005 | 0.0005 |
| N44 | 0.0256 | 0.0027 | 0.0026 | 0.0001 | 0.0011 | 0.9654 | 0.0010 | 0.0015 |
| N45 | 0.2654 | 0.1550 | 0.0129 | 0.0004 | 0.0011 | 0.5516 | 0.0014 | 0.0125 |
| N46 | 0.0012 | 0.0039 | 0.0024 | 0.0002 | 0.0004 | 0.9898 | 0.0012 | 0.0010 |
| N47 | 0.0009 | 0.0068 | 0.0057 | 0.0001 | 0.0011 | 0.9838 | 0.0010 | 0.0007 |
| N48 | 0.0001 | 0.0021 | 0.0020 | 0.0004 | 0.0005 | 0.9940 | 0.0004 | 0.0004 |
| N50 | 0.0039 | 0.0195 | 0.0475 | 0.0001 | 0.9202 | 0.0039 | 0.0016 | 0.0033 |
| N31 | 0.0030 | 0.0020 | 0.0013 | 0.0009 | 0.0005 | 0.9886 | 0.0005 | 0.0033 |
| R1 | 0.0017 | 0.6273 | 0.0214 | 0.0002 | 0.0006 | 0.0016 | 0.0008 | 0.3466 |
| R2 | 0.0015 | 0.0889 | 0.0023 | 0.0004 | 0.0013 | 0.0014 | 0.0529 | 0.8514 |
| R18 | 0.0037 | 0.0054 | 0.0281 | 0.0002 | 0.0015 | 0.0015 | 0.0004 | 0.9593 |
| R19 | 0.0016 | 0.0018 | 0.7642 | 0.0071 | 0.0208 | 0.0010 | 0.0078 | 0.1957 |
| R20 | 0.0026 | 0.0010 | 0.0044 | 0.0004 | 0.0063 | 0.0007 | 0.0003 | 0.9843 |
| R21 | 0.0073 | 0.0006 | 0.0042 | 0.0002 | 0.0375 | 0.0006 | 0.0012 | 0.9484 |
| R22 | 0.0017 | 0.1465 | 0.0055 | 0.0005 | 0.0312 | 0.3004 | 0.0005 | 0.5137 |
| R23 | 0.0937 | 0.0189 | 0.8025 | 0.0006 | 0.0032 | 0.0490 | 0.0047 | 0.0274 |
| R24 | 0.0033 | 0.0072 | 0.0035 | 0.0003 | 0.0078 | 0.0092 | 0.0003 | 0.9685 |
| R25 | 0.0199 | 0.0118 | 0.0047 | 0.0001 | 0.0059 | 0.0103 | 0.0019 | 0.9453 |
| R26 | 0.0054 | 0.0012 | 0.0043 | 0.0001 | 0.0164 | 0.0180 | 0.0005 | 0.9541 |
| R37 | 0.0422 | 0.0011 | 0.3714 | 0.0002 | 0.0016 | 0.0228 | 0.0002 | 0.5605 |
| R32 | 0.0169 | 0.0039 | 0.0027 | 0.0004 | 0.1174 | 0.0957 | 0.0002 | 0.7629 |
| R33 | 0.0016 | 0.0021 | 0.0019 | 0.0007 | 0.0038 | 0.0108 | 0.0014 | 0.9777 |
| R34 | 0.0117 | 0.0928 | 0.0055 | 0.0001 | 0.0456 | 0.0029 | 0.0008 | 0.8407 |
| R35 | 0.0062 | 0.0028 | 0.0071 | 0.0003 | 0.0787 | 0.0038 | 0.0027 | 0.8982 |
| R36 | 0.0005 | 0.0005 | 0.0029 | 0.0001 | 0.0033 | 0.0007 | 0.0003 | 0.9918 |
| R17 | 0.0520 | 0.0170 | 0.0009 | 0.0003 | 0.0007 | 0.0867 | 0.7809 | 0.0614 |
| R51 | 0.0012 | 0.0200 | 0.7370 | 0.0002 | 0.0027 | 0.0348 | 0.2030 | 0.0013 |
| R52 | 0.0006 | 0.0253 | 0.1024 | 0.0002 | 0.0011 | 0.0039 | 0.6678 | 0.1986 |

Table S2. continued

| Population code | Partial membership to each cluster | | | | | | | |
| --- | --- | --- | --- | --- | --- | --- | --- | --- |
|  | C1 | C2 | C3 | C4 | C5 | C6 | C7 | C8 |
| R28 | 0.0037 | 0.3831 | 0.5851 | 0.0001 | 0.0039 | 0.0035 | 0.0162 | 0.0043 |
| R29A | 0.1048 | 0.1169 | 0.4336 | 0.0004 | 0.0028 | 0.0794 | 0.0301 | 0.2323 |
| R29B | 0.0010 | 0.0525 | 0.8293 | 0.0005 | 0.0028 | 0.0972 | 0.0032 | 0.0138 |
| R3 | 0.0127 | 0.0838 | 0.7085 | 0.0005 | 0.0881 | 0.0911 | 0.0021 | 0.0132 |
| R4 | 0.0009 | 0.0056 | 0.9884 | 0.0001 | 0.0005 | 0.0016 | 0.0015 | 0.0013 |
| R5A | 0.0006 | 0.0081 | 0.9780 | 0.0004 | 0.0032 | 0.0011 | 0.0024 | 0.0063 |
| R5B | 0.0006 | 0.0018 | 0.9866 | 0.0003 | 0.0048 | 0.0011 | 0.0036 | 0.0014 |
| R6 | 0.0039 | 0.0129 | 0.8636 | 0.0005 | 0.0031 | 0.0061 | 0.0012 | 0.1088 |
| R7 | 0.0006 | 0.0032 | 0.9881 | 0.0002 | 0.0007 | 0.0016 | 0.0035 | 0.0020 |
| R8 | 0.0009 | 0.0093 | 0.9605 | 0.0003 | 0.0073 | 0.0022 | 0.0057 | 0.0141 |
| R9 | 0.0014 | 0.0149 | 0.9534 | 0.0003 | 0.0091 | 0.0067 | 0.0035 | 0.0107 |
| R11 | 0.0087 | 0.0788 | 0.0093 | 0.0019 | 0.0051 | 0.2011 | 0.6413 | 0.0540 |
| R12 | 0.0049 | 0.0998 | 0.6863 | 0.0003 | 0.0240 | 0.0734 | 0.0202 | 0.0911 |
| R13 | 0.0034 | 0.0053 | 0.8078 | 0.0005 | 0.0004 | 0.0542 | 0.0475 | 0.0809 |
| R14 | 0.0008 | 0.0024 | 0.9857 | 0.0012 | 0.0006 | 0.0015 | 0.0037 | 0.0041 |
| R15 | 0.0007 | 0.0058 | 0.8163 | 0.0067 | 0.0013 | 0.1549 | 0.0104 | 0.0040 |
| R16 | 0.0012 | 0.0084 | 0.9720 | 0.0006 | 0.0005 | 0.0133 | 0.0020 | 0.0020 |
| R38 | 0.0003 | 0.0040 | 0.9871 | 0.0004 | 0.0016 | 0.0024 | 0.0037 | 0.0007 |
| R39 | 0.0015 | 0.0009 | 0.4102 | 0.0002 | 0.0020 | 0.5576 | 0.0003 | 0.0273 |
| R40 | 0.0003 | 0.9656 | 0.0237 | 0.0023 | 0.0003 | 0.0013 | 0.0027 | 0.0039 |
| R41 | 0.0006 | 0.0044 | 0.8863 | 0.0017 | 0.0384 | 0.0642 | 0.0006 | 0.0039 |
| R42 | 0.0029 | 0.0049 | 0.9541 | 0.0008 | 0.0016 | 0.0329 | 0.0010 | 0.0016 |
| R44 | 0.0009 | 0.0033 | 0.2137 | 0.0009 | 0.0112 | 0.0158 | 0.7483 | 0.0059 |
| R46 | 0.0006 | 0.0244 | 0.9576 | 0.0018 | 0.0004 | 0.0061 | 0.0022 | 0.0069 |
| R47 | 0.0031 | 0.0095 | 0.0074 | 0.0013 | 0.0046 | 0.0179 | 0.0039 | 0.9523 |
| R48 | 0.0114 | 0.0087 | 0.5963 | 0.0006 | 0.1612 | 0.2118 | 0.0021 | 0.0082 |
| R49 | 0.0025 | 0.0449 | 0.2255 | 0.0005 | 0.0019 | 0.3871 | 0.0031 | 0.3347 |
| R30 | 0.0004 | 0.0013 | 0.1005 | 0.0002 | 0.0003 | 0.0011 | 0.0004 | 0.8958 |
| R31 | 0.0027 | 0.0044 | 0.1270 | 0.0003 | 0.0003 | 0.0197 | 0.0013 | 0.8443 |
| R53 | 0.0001 | 0.0011 | 0.0015 | 0.0002 | 0.0003 | 0.0013 | 0.9953 | 0.0002 |
| R54 | 0.0203 | 0.0012 | 0.9467 | 0.0005 | 0.0270 | 0.0020 | 0.0005 | 0.0017 |
| R55 | 0.0011 | 0.0022 | 0.9925 | 0.0011 | 0.0007 | 0.0005 | 0.0004 | 0.0014 |
| R56 | 0.0110 | 0.0060 | 0.4576 | 0.0009 | 0.0029 | 0.0026 | 0.0050 | 0.5141 |
| R58 | 0.0010 | 0.2264 | 0.5374 | 0.0014 | 0.0344 | 0.1807 | 0.0050 | 0.0138 |
| R59 | 0.0385 | 0.0014 | 0.8204 | 0.0064 | 0.0006 | 0.0006 | 0.0139 | 0.1180 |

Table S2. continued

| Population code | Partial membership to each cluster | | | | | | | |
| --- | --- | --- | --- | --- | --- | --- | --- | --- |
|  | C1 | C2 | C3 | C4 | C5 | C6 | C7 | C8 |
| R60 | 0.0009 | 0.0019 | 0.9915 | 0.0004 | 0.0010 | 0.0005 | 0.0009 | 0.0029 |
| R61 | 0.0011 | 0.0013 | 0.9760 | 0.0009 | 0.0100 | 0.0084 | 0.0006 | 0.0018 |
| R62 | 0.0025 | 0.0008 | 0.9775 | 0.0004 | 0.0099 | 0.0011 | 0.0015 | 0.0062 |
| R64 | 0.0002 | 0.0006 | 0.0012 | 0.0006 | 0.0001 | 0.0008 | 0.9964 | 0.0001 |
| R63 | 0.0003 | 0.0012 | 0.0035 | 0.0008 | 0.0004 | 0.0016 | 0.9920 | 0.0002 |
| R65 | 0.0002 | 0.0018 | 0.0028 | 0.0013 | 0.0002 | 0.0017 | 0.9914 | 0.0004 |
| R66 | 0.0000 | 0.0005 | 0.0007 | 0.0022 | 0.0001 | 0.0003 | 0.9961 | 0.0001 |
| R67 | 0.0000 | 0.0006 | 0.0008 | 0.0006 | 0.0001 | 0.0004 | 0.9972 | 0.0001 |
| M2 | 0.0000 | 0.0005 | 0.0002 | 0.9982 | 0.0000 | 0.0003 | 0.0007 | 0.0000 |
| M1 | 0.0001 | 0.0002 | 0.0004 | 0.9987 | 0.0000 | 0.0001 | 0.0003 | 0.0001 |
| M3 | 0.0000 | 0.0002 | 0.0007 | 0.9983 | 0.0001 | 0.0002 | 0.0005 | 0.0001 |
| M4 | 0.0000 | 0.0004 | 0.0004 | 0.9979 | 0.0001 | 0.0003 | 0.0008 | 0.0000 |
| M5 | 0.0000 | 0.0005 | 0.0009 | 0.9973 | 0.0000 | 0.0003 | 0.0008 | 0.0000 |
