# Supplementary material for: Local differentiation amidst extensive allele sharing in Oryza nivara and O. rufipogon
Source: Ecol Evol. 2013 Aug 1;3(9):3047–62. doi: 10.1002/ece3.689 (PMC3790550; doi:10.1002/ece3.689)
Supplement: Supplementary file 9 [file ece30003-3047-SD9.doc]

Table S3. Allele sizes and frequencies of 29 SSR markers in Asia Pacific *Oryza* series *Sativae*. Alleles that can distinguish all or certain population groups within a species are highlighted. Highly discriminating alleles are boldfaced.

| Marker/ Allele size (bp) | Allele frequencies | | | | | | | |
| --- | --- | --- | --- | --- | --- | --- | --- | --- |
|  | *O. nivara­­­­­­­­­­­­­­_________________* | | | | *O. rufipogon_______* | | | *O. meridionalis* |
|  | K1 | K2 | K3 | K4 | K5 | K6 | K7 | K8 |
| OSR13 |  |  |  |  |  |  |  |  |
| 106 | 0.000 | 0.071 | 0.000 | 0.007 | 0.080 | 0.000 | 0.000 | 0.000 |
| 108 | 0.000 | 0.000 | 0.000 | 0.000 | 0.029 | 0.000 | 0.034 | 0.000 |
| 110 | 0.000 | 0.000 | 0.000 | 0.000 | 0.051 | 0.000 | 0.114 | 0.000 |
| 112 | 0.000 | 0.000 | 0.000 | 0.185 | 0.304 | 0.125 | 0.045 | 0.000 |
| 114 | 0.152 | 0.086 | 0.125 | 0.089 | 0.159 | 0.146 | 0.341 | 0.000 |
| 116 | 0.109 | 0.000 | 0.194 | 0.116 | 0.051 | 0.217 | 0.386 | 1.000 |
| 118 | 0.000 | 0.000 | 0.181 | 0.000 | 0.000 | 0.192 | 0.000 | 0.000 |
| 120 | 0.207 | 0.214 | 0.028 | 0.014 | 0.051 | 0.000 | 0.000 | 0.000 |
| 122 | 0.000 | 0.486 | 0.000 | 0.247 | 0.007 | 0.133 | 0.080 | 0.000 |
| 124 | 0.000 | 0.143 | 0.000 | 0.000 | 0.000 | 0.000 | 0.000 | 0.000 |
| 126 | 0.163 | 0.000 | 0.000 | 0.068 | 0.000 | 0.000 | 0.000 | 0.000 |
| 128 | 0.000 | 0.000 | 0.042 | 0.137 | 0.022 | 0.000 | 0.000 | 0.000 |
| 130 | 0.152 | 0.000 | 0.000 | 0.082 | 0.014 | 0.042 | 0.000 | 0.000 |
| 132 | 0.109 | 0.000 | 0.118 | 0.000 | 0.000 | 0.050 | 0.000 | 0.000 |
| 134 | 0.109 | 0.000 | 0.118 | 0.034 | 0.232 | 0.092 | 0.000 | 0.000 |
| 136 | 0.000 | 0.000 | 0.104 | 0.000 | 0.000 | 0.000 | 0.000 | 0.000 |
| 138 | 0.000 | 0.000 | 0.000 | 0.000 | 0.000 | 0.004 | 0.000 | 0.000 |
| 140 | 0.000 | 0.000 | 0.090 | 0.007 | 0.000 | 0.000 | 0.000 | 0.000 |
| 142 | 0.000 | 0.000 | 0.000 | 0.014 | 0.000 | 0.000 | 0.000 | 0.000 |
|  |  |  |  |  |  |  |  |  |
| RM44 |  |  |  |  |  |  |  |  |
| 111 | 0.000 | 0.000 | 0.000 | 0.000 | 0.029 | 0.213 | 0.102 | 0.000 |
| 113 | 0.000 | 0.029 | 0.000 | 0.000 | 0.036 | 0.317 | 0.000 | 0.000 |
| 117 | 0.000 | 0.000 | 0.000 | 0.000 | 0.094 | 0.008 | 0.330 | 0.000 |
| 119 | 0.000 | 0.143 | 0.097 | 0.000 | 0.080 | 0.025 | 0.045 | 0.083 |
| 121 | 0.370 | 0.000 | 0.042 | 0.233 | 0.254 | 0.058 | 0.000 | 0.208 |
| 123 | 0.000 | 0.057 | 0.028 | 0.068 | 0.196 | 0.050 | 0.295 | 0.167 |
| 125 | 0.065 | 0.143 | 0.014 | 0.151 | 0.036 | 0.104 | 0.091 | 0.000 |
| 127 | 0.000 | 0.486 | 0.222 | 0.068 | 0.029 | 0.075 | 0.000 | 0.125 |
| 129 | 0.554 | 0.143 | 0.250 | 0.308 | 0.029 | 0.088 | 0.068 | 0.000 |
| 131 | 0.000 | 0.000 | 0.250 | 0.000 | 0.159 | 0.000 | 0.000 | 0.000 |
| 133 | 0.000 | 0.000 | 0.097 | 0.110 | 0.058 | 0.000 | 0.068 | 0.000 |
| 135 | 0.000 | 0.000 | 0.000 | 0.000 | 0.000 | 0.000 | 0.000 | 0.208 |
| 137 | 0.000 | 0.000 | 0.000 | 0.000 | 0.000 | 0.017 | 0.000 | 0.000 |

Table S3. continued

| Marker/ Allele size (bp) | Allele frequencies | | | | | | | |
| --- | --- | --- | --- | --- | --- | --- | --- | --- |
|  | *O. nivara­­­­­­­­­­­­­­_________________* | | | | *O. rufipogon_______* | | | *O. meridionalis* |
|  | K1 | K2 | K3 | K4 | K5 | K6 | K7 | K8 |
| 139 | 0.000 | 0.000 | 0.000 | 0.055 | 0.000 | 0.046 | 0.000 | 0.000 |
| 141 | 0.000 | 0.000 | 0.000 | 0.000 | 0.000 | 0.000 | 0.000 | 0.208 |
| 143 | 0.011 | 0.000 | 0.000 | 0.000 | 0.000 | 0.000 | 0.000 | 0.000 |
| 149 | 0.000 | 0.000 | 0.000 | 0.007 | 0.000 | 0.000 | 0.000 | 0.000 |
|  |  |  |  |  |  |  |  |  |
| RM118 |  |  |  |  |  |  |  |  |
| 153 | 0.000 | 0.000 | 0.000 | 0.000 | 0.000 | 0.000 | 0.000 | 0.125 |
| 171 | 0.000 | 0.000 | 0.014 | 0.000 | 0.000 | 0.000 | 0.000 | 0.875 |
| 173 | 0.000 | 0.000 | 0.000 | 0.000 | 0.000 | 0.000 | 0.466 | 0.000 |
| 175 | 0.422 | 0.743 | 0.569 | 0.178 | 0.565 | 0.447 | 0.227 | 0.000 |
| 177 | 0.000 | 0.143 | 0.000 | 0.014 | 0.101 | 0.148 | 0.000 | 0.000 |
| 179 | 0.556 | 0.114 | 0.417 | 0.630 | 0.246 | 0.340 | 0.307 | 0.000 |
| 181 | 0.000 | 0.000 | 0.000 | 0.041 | 0.087 | 0.066 | 0.000 | 0.000 |
| 185 | 0.022 | 0.000 | 0.000 | 0.082 | 0.000 | 0.000 | 0.000 | 0.000 |
| 195 | 0.000 | 0.000 | 0.000 | 0.055 | 0.000 | 0.000 | 0.000 | 0.000 |
|  |  |  |  |  |  |  |  |  |
| RM124 |  |  |  |  |  |  |  |  |
| 280 | 0.043 | 0.000 | 0.000 | 0.000 | 0.000 | 0.000 | 0.000 | 0.000 |
| 282 | 0.217 | 0.000 | 0.000 | 0.192 | 0.000 | 0.033 | 0.000 | 0.000 |
| 284 | 0.739 | 0.943 | 0.806 | 0.753 | 0.812 | 0.852 | 0.227 | 0.000 |
| 286 | 0.000 | 0.057 | 0.194 | 0.055 | 0.188 | 0.025 | 0.682 | 0.000 |
| 288 | 0.000 | 0.000 | 0.000 | 0.000 | 0.000 | 0.090 | 0.045 | 0.000 |
| 290 | 0.000 | 0.000 | 0.000 | 0.000 | 0.000 | 0.000 | 0.023 | 0.000 |
| 296 | 0.000 | 0.000 | 0.000 | 0.000 | 0.000 | 0.000 | 0.023 | 0.000 |
| **298** | 0.000 | 0.000 | 0.000 | 0.000 | 0.000 | 0.000 | 0.000 | 1.000 |
|  |  |  |  |  |  |  |  |  |
| RM125 |  |  |  |  |  |  |  |  |
| 129 | 0.000 | 0.000 | 0.000 | 0.068 | 0.043 | 0.000 | 0.000 | 0.000 |
| 132 | 0.000 | 0.029 | 0.000 | 0.000 | 0.022 | 0.116 | 0.000 | 0.000 |
| 135 | 0.000 | 0.000 | 0.000 | 0.000 | 0.000 | 0.198 | 0.091 | 1.000 |
| 138 | 0.109 | 0.529 | 0.028 | 0.000 | 0.630 | 0.012 | 0.000 | 0.000 |
| 141 | 0.217 | 0.000 | 0.313 | 0.740 | 0.000 | 0.273 | 0.000 | 0.000 |
| 144 | 0.196 | 0.000 | 0.097 | 0.000 | 0.254 | 0.219 | 0.773 | 0.000 |
| 147 | 0.000 | 0.088 | 0.125 | 0.007 | 0.000 | 0.000 | 0.045 | 0.000 |
| 150 | 0.000 | 0.294 | 0.000 | 0.000 | 0.036 | 0.116 | 0.091 | 0.000 |
| 153 | 0.370 | 0.059 | 0.181 | 0.068 | 0.000 | 0.041 | 0.000 | 0.000 |
| 156 | 0.109 | 0.000 | 0.257 | 0.116 | 0.014 | 0.017 | 0.000 | 0.000 |

Table S3. continued

| Marker/ Allele size (bp) | Allele frequencies | | | | | | | |
| --- | --- | --- | --- | --- | --- | --- | --- | --- |
|  | *O. nivara­­­­­­­­­­­­­­_________________* | | | | *O. rufipogon_______* | | | *O. meridionalis* |
|  | K1 | K2 | K3 | K4 | K5 | K6 | K7 | K8 |
| 162 | 0.000 | 0.000 | 0.000 | 0.000 | 0.000 | 0.008 | 0.000 | 0.000 |
|  |  |  |  |  |  |  |  |  |
| RM133 |  |  |  |  |  |  |  |  |
| 243 | 0.413 | 0.971 | 0.882 | 0.514 | 0.174 | 0.246 | 0.205 | 0.208 |
| 245 | 0.152 | 0.000 | 0.056 | 0.375 | 0.652 | 0.324 | 0.534 | 0.792 |
| 247 | 0.435 | 0.000 | 0.014 | 0.014 | 0.174 | 0.225 | 0.023 | 0.000 |
| 249 | 0.000 | 0.014 | 0.049 | 0.097 | 0.000 | 0.164 | 0.193 | 0.000 |
| 251 | 0.000 | 0.000 | 0.000 | 0.000 | 0.000 | 0.008 | 0.045 | 0.000 |
| 255 | 0.000 | 0.014 | 0.000 | 0.000 | 0.000 | 0.033 | 0.000 | 0.000 |
|  |  |  |  |  |  |  |  |  |
| RM152 |  |  |  |  |  |  |  |  |
| 149 | 0.380 | 0.000 | 0.076 | 0.055 | 0.087 | 0.008 | 0.000 | 0.000 |
| 152 | 0.000 | 0.000 | 0.125 | 0.205 | 0.181 | 0.016 | 0.000 | 0.000 |
| 155 | 0.087 | 0.000 | 0.000 | 0.014 | 0.000 | 0.000 | 0.011 | 1.000 |
| 158 | 0.174 | 0.857 | 0.104 | 0.000 | 0.391 | 0.541 | 0.284 | 0.000 |
| 161 | 0.359 | 0.143 | 0.611 | 0.658 | 0.283 | 0.373 | 0.659 | 0.000 |
| 164 | 0.000 | 0.000 | 0.000 | 0.000 | 0.029 | 0.000 | 0.000 | 0.000 |
| 167 | 0.000 | 0.000 | 0.076 | 0.027 | 0.014 | 0.053 | 0.000 | 0.000 |
| 170 | 0.000 | 0.000 | 0.007 | 0.041 | 0.014 | 0.000 | 0.045 | 0.000 |
| 173 | 0.000 | 0.000 | 0.000 | 0.000 | 0.000 | 0.008 | 0.000 | 0.000 |
|  |  |  |  |  |  |  |  |  |
| RM154 |  |  |  |  |  |  |  |  |
| 178 | 0.000 | 0.000 | 0.000 | 0.000 | 0.015 | 0.020 | 0.023 | 0.000 |
| 180 | 0.000 | 0.000 | 0.000 | 0.000 | 0.000 | 0.012 | 0.091 | 0.000 |
| 182 | 0.000 | 0.000 | 0.000 | 0.096 | 0.067 | 0.012 | 0.170 | 0.000 |
| 184 | 0.087 | 0.000 | 0.583 | 0.260 | 0.082 | 0.389 | 0.114 | 0.208 |
| 186 | 0.239 | 0.171 | 0.236 | 0.068 | 0.164 | 0.160 | 0.000 | 0.771 |
| 188 | 0.000 | 0.000 | 0.069 | 0.164 | 0.030 | 0.020 | 0.011 | 0.000 |
| 189 | 0.000 | 0.000 | 0.000 | 0.000 | 0.007 | 0.000 | 0.000 | 0.000 |
| 190 | 0.652 | 0.829 | 0.000 | 0.260 | 0.112 | 0.082 | 0.170 | 0.000 |
| 192 | 0.022 | 0.000 | 0.069 | 0.000 | 0.045 | 0.000 | 0.023 | 0.000 |
| 194 | 0.000 | 0.000 | 0.014 | 0.000 | 0.052 | 0.020 | 0.193 | 0.000 |
| 196 | 0.000 | 0.000 | 0.000 | 0.000 | 0.000 | 0.012 | 0.205 | 0.000 |
| 198 | 0.000 | 0.000 | 0.000 | 0.000 | 0.097 | 0.004 | 0.000 | 0.000 |
| 200 | 0.000 | 0.000 | 0.028 | 0.068 | 0.037 | 0.094 | 0.000 | 0.000 |
| 202 | 0.000 | 0.000 | 0.000 | 0.000 | 0.022 | 0.090 | 0.000 | 0.021 |
| 204 | 0.000 | 0.000 | 0.000 | 0.000 | 0.090 | 0.000 | 0.000 | 0.000 |

Table S3. continued

| Marker/ Allele size (bp) | Allele frequencies | | | | | | | |
| --- | --- | --- | --- | --- | --- | --- | --- | --- |
|  | *O. nivara­­­­­­­­­­­­­­_________________* | | | | *O. rufipogon_______* | | | *O. meridionalis* |
|  | K1 | K2 | K3 | K4 | K5 | K6 | K7 | K8 |
| 206 | 0.000 | 0.000 | 0.000 | 0.068 | 0.015 | 0.029 | 0.000 | 0.000 |
| 208 | 0.000 | 0.000 | 0.000 | 0.000 | 0.075 | 0.000 | 0.000 | 0.000 |
| 210 | 0.000 | 0.000 | 0.000 | 0.014 | 0.015 | 0.000 | 0.000 | 0.000 |
| 212 | 0.000 | 0.000 | 0.000 | 0.000 | 0.000 | 0.020 | 0.000 | 0.000 |
| 216 | 0.000 | 0.000 | 0.000 | 0.000 | 0.022 | 0.000 | 0.000 | 0.000 |
| 218 | 0.000 | 0.000 | 0.000 | 0.000 | 0.045 | 0.000 | 0.000 | 0.000 |
| 224 | 0.000 | 0.000 | 0.000 | 0.000 | 0.000 | 0.033 | 0.000 | 0.000 |
| 228 | 0.000 | 0.000 | 0.000 | 0.000 | 0.007 | 0.000 | 0.000 | 0.000 |
|  |  |  |  |  |  |  |  |  |
| RM161 |  |  |  |  |  |  |  |  |
| 171 | 0.000 | 0.000 | 0.000 | 0.000 | 0.000 | 0.025 | 0.000 | 0.000 |
| 173 | 0.000 | 0.000 | 0.056 | 0.000 | 0.457 | 0.434 | 0.000 | 0.000 |
| 175 | 0.000 | 0.000 | 0.000 | 0.000 | 0.000 | 0.000 | 0.000 | 0.125 |
| 177 | 0.000 | 0.000 | 0.000 | 0.000 | 0.000 | 0.033 | 0.443 | 0.583 |
| 179 | 0.000 | 0.000 | 0.063 | 0.000 | 0.014 | 0.017 | 0.114 | 0.292 |
| 181 | 0.587 | 0.286 | 0.148 | 0.123 | 0.000 | 0.008 | 0.000 | 0.000 |
| 183 | 0.000 | 0.714 | 0.169 | 0.534 | 0.130 | 0.099 | 0.330 | 0.000 |
| 185 | 0.217 | 0.000 | 0.148 | 0.137 | 0.188 | 0.140 | 0.114 | 0.000 |
| 187 | 0.000 | 0.000 | 0.000 | 0.137 | 0.138 | 0.025 | 0.000 | 0.000 |
| 189 | 0.000 | 0.000 | 0.028 | 0.000 | 0.058 | 0.033 | 0.000 | 0.000 |
| 191 | 0.000 | 0.000 | 0.387 | 0.000 | 0.000 | 0.116 | 0.000 | 0.000 |
| 193 | 0.000 | 0.000 | 0.000 | 0.000 | 0.000 | 0.033 | 0.000 | 0.000 |
| 197 | 0.196 | 0.000 | 0.000 | 0.000 | 0.000 | 0.000 | 0.000 | 0.000 |
| 199 | 0.000 | 0.000 | 0.000 | 0.068 | 0.014 | 0.037 | 0.000 | 0.000 |
|  |  |  |  |  |  |  |  |  |
| RM162 |  |  |  |  |  |  |  |  |
| 220 | 0.065 | 0.000 | 0.000 | 0.000 | 0.000 | 0.000 | 0.000 | 0.000 |
| 222 | 0.000 | 0.000 | 0.000 | 0.000 | 0.043 | 0.000 | 0.000 | 0.000 |
| 224 | 0.043 | 0.000 | 0.000 | 0.068 | 0.007 | 0.008 | 0.000 | 0.000 |
| 226 | 0.000 | 0.000 | 0.014 | 0.000 | 0.051 | 0.033 | 0.000 | 0.000 |
| 228 | 0.000 | 0.000 | 0.056 | 0.014 | 0.000 | 0.045 | 0.000 | 0.958 |
| 230 | 0.000 | 0.686 | 0.299 | 0.000 | 0.181 | 0.120 | 0.182 | 0.000 |
| 232 | 0.098 | 0.000 | 0.139 | 0.123 | 0.232 | 0.186 | 0.000 | 0.000 |
| 234 | 0.413 | 0.000 | 0.326 | 0.425 | 0.116 | 0.244 | 0.455 | 0.000 |
| 236 | 0.033 | 0.029 | 0.000 | 0.068 | 0.036 | 0.136 | 0.341 | 0.000 |
| 238 | 0.043 | 0.143 | 0.042 | 0.219 | 0.080 | 0.025 | 0.000 | 0.000 |
| 240 | 0.000 | 0.143 | 0.118 | 0.000 | 0.007 | 0.116 | 0.000 | 0.000 |

Table S3. continued

| Marker/ Allele size (bp) | Allele frequencies | | | | | | | |
| --- | --- | --- | --- | --- | --- | --- | --- | --- |
|  | *O. nivara­­­­­­­­­­­­­­_________________* | | | | *O. rufipogon_______* | | | *O. meridionalis* |
|  | K1 | K2 | K3 | K4 | K5 | K6 | K7 | K8 |
| 242 | 0.000 | 0.000 | 0.000 | 0.000 | 0.101 | 0.021 | 0.000 | 0.000 |
| 244 | 0.000 | 0.000 | 0.000 | 0.000 | 0.101 | 0.000 | 0.000 | 0.000 |
| 246 | 0.000 | 0.000 | 0.000 | 0.000 | 0.000 | 0.008 | 0.000 | 0.000 |
| 250 | 0.000 | 0.000 | 0.007 | 0.000 | 0.000 | 0.041 | 0.000 | 0.000 |
| 256 | 0.000 | 0.000 | 0.000 | 0.000 | 0.014 | 0.008 | 0.000 | 0.000 |
| 260 | 0.000 | 0.000 | 0.000 | 0.000 | 0.000 | 0.000 | 0.023 | 0.000 |
| 262 | 0.304 | 0.000 | 0.000 | 0.027 | 0.000 | 0.000 | 0.000 | 0.000 |
| 264 | 0.000 | 0.000 | 0.000 | 0.000 | 0.014 | 0.000 | 0.000 | 0.000 |
| 266 | 0.000 | 0.000 | 0.000 | 0.055 | 0.000 | 0.000 | 0.000 | 0.042 |
| 268 | 0.000 | 0.000 | 0.000 | 0.000 | 0.014 | 0.008 | 0.000 | 0.000 |
|  |  |  |  |  |  |  |  |  |
| RM215 |  |  |  |  |  |  |  |  |
| 146 | 0.000 | 0.029 | 0.000 | 0.000 | 0.000 | 0.000 | 0.000 | 0.000 |
| 148 | 0.000 | 0.000 | 0.014 | 0.000 | 0.022 | 0.020 | 0.000 | 0.917 |
| 150 | 0.000 | 0.000 | 0.000 | 0.000 | 0.036 | 0.000 | 0.023 | 0.000 |
| 152 | 0.000 | 0.486 | 0.000 | 0.034 | 0.051 | 0.127 | 0.000 | 0.042 |
| 154 | 0.022 | 0.086 | 0.097 | 0.082 | 0.145 | 0.205 | 0.034 | 0.000 |
| 156 | 0.000 | 0.200 | 0.000 | 0.014 | 0.174 | 0.184 | 0.659 | 0.000 |
| 158 | 0.000 | 0.029 | 0.333 | 0.034 | 0.174 | 0.107 | 0.057 | 0.000 |
| 160 | 0.207 | 0.000 | 0.042 | 0.055 | 0.130 | 0.160 | 0.000 | 0.042 |
| 162 | 0.326 | 0.000 | 0.250 | 0.212 | 0.065 | 0.045 | 0.068 | 0.000 |
| 164 | 0.185 | 0.000 | 0.042 | 0.185 | 0.094 | 0.090 | 0.034 | 0.000 |
| 166 | 0.109 | 0.029 | 0.069 | 0.288 | 0.051 | 0.041 | 0.080 | 0.000 |
| 168 | 0.130 | 0.000 | 0.042 | 0.082 | 0.014 | 0.008 | 0.000 | 0.000 |
| 170 | 0.022 | 0.143 | 0.014 | 0.000 | 0.007 | 0.004 | 0.045 | 0.000 |
| 172 | 0.000 | 0.000 | 0.000 | 0.014 | 0.014 | 0.008 | 0.000 | 0.000 |
| 176 | 0.000 | 0.000 | 0.083 | 0.000 | 0.000 | 0.000 | 0.000 | 0.000 |
| 178 | 0.000 | 0.000 | 0.000 | 0.000 | 0.022 | 0.000 | 0.000 | 0.000 |
| 184 | 0.000 | 0.000 | 0.014 | 0.000 | 0.000 | 0.000 | 0.000 | 0.000 |
|  |  |  |  |  |  |  |  |  |
| RM237 |  |  |  |  |  |  |  |  |
| 120 | 0.000 | 0.000 | 0.000 | 0.000 | 0.029 | 0.000 | 0.000 | 0.000 |
| 126 | 0.000 | 0.000 | 0.000 | 0.000 | 0.000 | 0.000 | 0.000 | 0.065 |
| 128 | 0.000 | 0.057 | 0.007 | 0.014 | 0.000 | 0.000 | 0.000 | 0.000 |
| 130 | 0.000 | 0.029 | 0.083 | 0.000 | 0.138 | 0.020 | 0.034 | 0.000 |
| 132 | 0.000 | 0.000 | 0.021 | 0.021 | 0.217 | 0.000 | 0.136 | 0.000 |
| 134 | 0.022 | 0.029 | 0.042 | 0.027 | 0.094 | 0.057 | 0.000 | 0.000 |

Table S3. continued

| Marker/ Allele size (bp) | Allele frequencies | | | | | | | |
| --- | --- | --- | --- | --- | --- | --- | --- | --- |
|  | *O. nivara­­­­­­­­­­­­­­_________________* | | | | *O. rufipogon_______* | | | *O. meridionalis* |
|  | K1 | K2 | K3 | K4 | K5 | K6 | K7 | K8 |
| 136 | 0.000 | 0.000 | 0.014 | 0.000 | 0.000 | 0.049 | 0.250 | 0.000 |
| 138 | 0.000 | 0.000 | 0.000 | 0.000 | 0.014 | 0.160 | 0.068 | 0.022 |
| 140 | 0.783 | 0.114 | 0.139 | 0.000 | 0.203 | 0.061 | 0.000 | 0.000 |
| 142 | 0.109 | 0.000 | 0.056 | 0.082 | 0.043 | 0.090 | 0.102 | 0.065 |
| 144 | 0.065 | 0.000 | 0.292 | 0.164 | 0.072 | 0.012 | 0.000 | 0.000 |
| 146 | 0.000 | 0.000 | 0.028 | 0.082 | 0.000 | 0.053 | 0.034 | 0.000 |
| 148 | 0.022 | 0.057 | 0.014 | 0.233 | 0.051 | 0.000 | 0.000 | 0.000 |
| 150 | 0.000 | 0.143 | 0.118 | 0.349 | 0.036 | 0.172 | 0.136 | 0.543 |
| 152 | 0.000 | 0.000 | 0.000 | 0.000 | 0.014 | 0.090 | 0.000 | 0.000 |
| 154 | 0.000 | 0.486 | 0.097 | 0.000 | 0.022 | 0.041 | 0.125 | 0.304 |
| 156 | 0.000 | 0.000 | 0.014 | 0.027 | 0.000 | 0.070 | 0.000 | 0.000 |
| 158 | 0.000 | 0.086 | 0.028 | 0.000 | 0.029 | 0.041 | 0.091 | 0.000 |
| 160 | 0.000 | 0.000 | 0.000 | 0.000 | 0.029 | 0.016 | 0.000 | 0.000 |
| 166 | 0.000 | 0.000 | 0.000 | 0.000 | 0.000 | 0.004 | 0.000 | 0.000 |
| 168 | 0.000 | 0.000 | 0.000 | 0.000 | 0.000 | 0.029 | 0.000 | 0.000 |
| 170 | 0.000 | 0.000 | 0.049 | 0.000 | 0.007 | 0.000 | 0.023 | 0.000 |
| 176 | 0.000 | 0.000 | 0.000 | 0.000 | 0.000 | 0.033 | 0.000 | 0.000 |
|  |  |  |  |  |  |  |  |  |
| RM271 |  |  |  |  |  |  |  |  |
| 104 | 0.000 | 0.114 | 0.160 | 0.055 | 0.000 | 0.144 | 0.000 | 0.208 |
| 106 | 0.000 | 0.000 | 0.000 | 0.041 | 0.125 | 0.021 | 0.000 | 0.333 |
| 108 | 0.000 | 0.000 | 0.000 | 0.000 | 0.199 | 0.030 | 0.000 | 0.000 |
| 110 | 0.435 | 0.014 | 0.174 | 0.048 | 0.162 | 0.047 | 0.000 | 0.000 |
| 112 | 0.174 | 0.229 | 0.201 | 0.137 | 0.000 | 0.042 | 0.068 | 0.000 |
| 114 | 0.011 | 0.000 | 0.201 | 0.123 | 0.059 | 0.038 | 0.182 | 0.000 |
| 116 | 0.000 | 0.029 | 0.125 | 0.000 | 0.000 | 0.008 | 0.443 | 0.083 |
| 118 | 0.054 | 0.014 | 0.007 | 0.315 | 0.191 | 0.322 | 0.023 | 0.208 |
| 120 | 0.000 | 0.000 | 0.000 | 0.000 | 0.000 | 0.123 | 0.000 | 0.000 |
| 122 | 0.207 | 0.000 | 0.000 | 0.000 | 0.029 | 0.034 | 0.000 | 0.167 |
| 124 | 0.120 | 0.000 | 0.000 | 0.253 | 0.029 | 0.004 | 0.159 | 0.000 |
| 126 | 0.000 | 0.600 | 0.021 | 0.000 | 0.000 | 0.008 | 0.000 | 0.000 |
| 128 | 0.000 | 0.000 | 0.049 | 0.000 | 0.015 | 0.017 | 0.000 | 0.000 |
| 130 | 0.000 | 0.000 | 0.007 | 0.000 | 0.088 | 0.068 | 0.000 | 0.000 |
| 132 | 0.000 | 0.000 | 0.000 | 0.000 | 0.000 | 0.008 | 0.011 | 0.000 |
| 134 | 0.000 | 0.000 | 0.000 | 0.000 | 0.000 | 0.030 | 0.000 | 0.000 |
| 136 | 0.000 | 0.000 | 0.000 | 0.000 | 0.000 | 0.034 | 0.114 | 0.000 |
| 138 | 0.000 | 0.000 | 0.000 | 0.000 | 0.074 | 0.000 | 0.000 | 0.000 |

Table S3. continued

| Marker/ Allele size (bp) | Allele frequencies | | | | | | | |
| --- | --- | --- | --- | --- | --- | --- | --- | --- |
|  | *O. nivara­­­­­­­­­­­­­­_________________* | | | | *O. rufipogon_______* | | | *O. meridionalis* |
|  | K1 | K2 | K3 | K4 | K5 | K6 | K7 | K8 |
| 140 | 0.000 | 0.000 | 0.056 | 0.000 | 0.000 | 0.000 | 0.000 | 0.000 |
| 142 | 0.000 | 0.000 | 0.000 | 0.027 | 0.015 | 0.000 | 0.000 | 0.000 |
| 144 | 0.000 | 0.000 | 0.000 | 0.000 | 0.015 | 0.000 | 0.000 | 0.000 |
| 146 | 0.000 | 0.000 | 0.000 | 0.000 | 0.000 | 0.013 | 0.000 | 0.000 |
| 156 | 0.000 | 0.000 | 0.000 | 0.000 | 0.000 | 0.008 | 0.000 | 0.000 |
|  |  |  |  |  |  |  |  |  |
| RM277 |  |  |  |  |  |  |  |  |
| 128 | 0.000 | 0.000 | 0.014 | 0.000 | 0.000 | 0.000 | 0.000 | 0.000 |
| 130 | 0.000 | 0.057 | 0.000 | 0.000 | 0.000 | 0.000 | 0.000 | 0.000 |
| 132 | 0.000 | 0.000 | 0.000 | 0.027 | 0.000 | 0.020 | 0.000 | 0.000 |
| 134 | 0.000 | 0.000 | 0.458 | 0.082 | 0.587 | 0.176 | 0.193 | 1.000 |
| 136 | 0.000 | 0.000 | 0.250 | 0.315 | 0.000 | 0.402 | 0.000 | 0.000 |
| 138 | 0.848 | 0.800 | 0.222 | 0.466 | 0.341 | 0.250 | 0.239 | 0.000 |
| 140 | 0.152 | 0.143 | 0.042 | 0.110 | 0.072 | 0.152 | 0.455 | 0.000 |
| 142 | 0.000 | 0.000 | 0.014 | 0.000 | 0.000 | 0.000 | 0.114 | 0.000 |
|  |  |  |  |  |  |  |  |  |
| RM283 |  |  |  |  |  |  |  |  |
| 151 | 0.000 | 0.029 | 0.000 | 0.000 | 0.015 | 0.004 | 0.000 | 0.000 |
| 153 | 0.000 | 0.000 | 0.000 | 0.000 | 0.000 | 0.008 | 0.114 | 0.000 |
| 155 | 0.000 | 0.000 | 0.000 | 0.021 | 0.044 | 0.000 | 0.000 | 0.000 |
| 157 | 0.000 | 0.400 | 0.014 | 0.000 | 0.000 | 0.109 | 0.000 | 0.000 |
| 159 | 0.000 | 0.114 | 0.000 | 0.000 | 0.029 | 0.050 | 0.000 | 0.000 |
| 161 | 0.000 | 0.000 | 0.000 | 0.127 | 0.015 | 0.084 | 0.307 | 0.000 |
| 163 | 0.023 | 0.000 | 0.153 | 0.070 | 0.015 | 0.034 | 0.193 | 0.000 |
| 165 | 0.000 | 0.029 | 0.000 | 0.014 | 0.081 | 0.042 | 0.000 | 0.000 |
| 167 | 0.114 | 0.000 | 0.028 | 0.000 | 0.000 | 0.000 | 0.000 | 0.000 |
| 169 | 0.114 | 0.171 | 0.000 | 0.085 | 0.140 | 0.239 | 0.000 | 0.167 |
| 171 | 0.205 | 0.143 | 0.486 | 0.056 | 0.316 | 0.134 | 0.239 | 0.000 |
| 173 | 0.000 | 0.114 | 0.069 | 0.049 | 0.000 | 0.076 | 0.000 | 0.000 |
| 175 | 0.205 | 0.000 | 0.028 | 0.155 | 0.250 | 0.021 | 0.000 | 0.625 |
| 177 | 0.182 | 0.000 | 0.000 | 0.127 | 0.059 | 0.084 | 0.034 | 0.208 |
| 179 | 0.068 | 0.000 | 0.181 | 0.028 | 0.000 | 0.008 | 0.000 | 0.000 |
| 181 | 0.000 | 0.000 | 0.042 | 0.014 | 0.000 | 0.042 | 0.114 | 0.000 |
| 183 | 0.091 | 0.000 | 0.000 | 0.000 | 0.000 | 0.046 | 0.000 | 0.000 |
| 185 | 0.000 | 0.000 | 0.000 | 0.183 | 0.000 | 0.008 | 0.000 | 0.000 |
| 187 | 0.000 | 0.000 | 0.000 | 0.070 | 0.037 | 0.000 | 0.000 | 0.000 |
| 197 | 0.000 | 0.000 | 0.000 | 0.000 | 0.000 | 0.008 | 0.000 | 0.000 |

Table S3. continued

| Marker/ Allele size (bp) | Allele frequencies | | | | | | | |
| --- | --- | --- | --- | --- | --- | --- | --- | --- |
|  | *O. nivara­­­­­­­­­­­­­­_________________* | | | | *O. rufipogon_______* | | | *O. meridionalis* |
|  | K1 | K2 | K3 | K4 | K5 | K6 | K7 | K8 |
|  |  |  |  |  |  |  |  |  |
| RM284 |  |  |  |  |  |  |  |  |
| 159 | 0.000 | 0.186 | 0.000 | 0.000 | 0.000 | 0.107 | 0.000 | 0.000 |
| 161 | 0.957 | 0.000 | 0.542 | 0.795 | 0.841 | 0.459 | 0.182 | 1.000 |
| 163 | 0.022 | 0.143 | 0.319 | 0.068 | 0.072 | 0.266 | 0.580 | 0.000 |
| 165 | 0.022 | 0.671 | 0.042 | 0.068 | 0.029 | 0.127 | 0.159 | 0.000 |
| 167 | 0.000 | 0.000 | 0.000 | 0.068 | 0.051 | 0.029 | 0.080 | 0.000 |
| 169 | 0.000 | 0.000 | 0.097 | 0.000 | 0.000 | 0.008 | 0.000 | 0.000 |
| 173 | 0.000 | 0.000 | 0.000 | 0.000 | 0.007 | 0.000 | 0.000 | 0.000 |
| 177 | 0.000 | 0.000 | 0.000 | 0.000 | 0.000 | 0.004 | 0.000 | 0.000 |
|  |  |  |  |  |  |  |  |  |
| RM316 |  |  |  |  |  |  |  |  |
| **134** | 0.000 | 0.000 | 0.000 | 0.000 | 0.000 | 0.000 | 0.000 | 1.000 |
| 180 | 0.000 | 0.000 | 0.000 | 0.000 | 0.000 | 0.025 | 0.000 | 0.000 |
| 186 | 0.000 | 0.000 | 0.000 | 0.000 | 0.000 | 0.008 | 0.023 | 0.000 |
| 187 | 0.000 | 0.000 | 0.000 | 0.000 | 0.000 | 0.041 | 0.000 | 0.000 |
| 215 | 0.000 | 0.000 | 0.000 | 0.000 | 0.051 | 0.000 | 0.000 | 0.000 |
| 216 | 0.011 | 0.000 | 0.000 | 0.000 | 0.000 | 0.000 | 0.000 | 0.000 |
| 217 | 0.000 | 0.000 | 0.000 | 0.000 | 0.000 | 0.029 | 0.023 | 0.000 |
| 218 | 0.000 | 0.114 | 0.083 | 0.397 | 0.181 | 0.182 | 0.291 | 0.000 |
| 219 | 0.000 | 0.000 | 0.000 | 0.205 | 0.000 | 0.000 | 0.000 | 0.000 |
| 220 | 0.000 | 0.000 | 0.000 | 0.068 | 0.000 | 0.008 | 0.105 | 0.000 |
| 221 | 0.000 | 0.000 | 0.000 | 0.000 | 0.000 | 0.000 | 0.070 | 0.000 |
| 222 | 0.000 | 0.000 | 0.472 | 0.082 | 0.022 | 0.091 | 0.023 | 0.000 |
| 223 | 0.000 | 0.000 | 0.000 | 0.000 | 0.000 | 0.017 | 0.000 | 0.000 |
| 224 | 0.989 | 0.029 | 0.069 | 0.123 | 0.000 | 0.037 | 0.000 | 0.000 |
| 225 | 0.000 | 0.000 | 0.014 | 0.000 | 0.043 | 0.004 | 0.000 | 0.000 |
| 226 | 0.000 | 0.143 | 0.000 | 0.000 | 0.130 | 0.012 | 0.047 | 0.000 |
| 227 | 0.000 | 0.543 | 0.000 | 0.000 | 0.116 | 0.074 | 0.000 | 0.000 |
| 228 | 0.000 | 0.000 | 0.007 | 0.000 | 0.000 | 0.025 | 0.174 | 0.000 |
| 229 | 0.000 | 0.143 | 0.000 | 0.000 | 0.000 | 0.004 | 0.000 | 0.000 |
| 230 | 0.000 | 0.000 | 0.139 | 0.000 | 0.225 | 0.182 | 0.163 | 0.000 |
| 231 | 0.000 | 0.000 | 0.000 | 0.000 | 0.000 | 0.017 | 0.000 | 0.000 |
| 233 | 0.000 | 0.029 | 0.000 | 0.123 | 0.014 | 0.025 | 0.000 | 0.000 |
| 235 | 0.000 | 0.000 | 0.146 | 0.000 | 0.007 | 0.099 | 0.000 | 0.000 |
| 236 | 0.000 | 0.000 | 0.000 | 0.000 | 0.036 | 0.000 | 0.000 | 0.000 |
| 237 | 0.000 | 0.000 | 0.014 | 0.000 | 0.014 | 0.008 | 0.000 | 0.000 |

Table S3. continued

| Marker/ Allele size (bp) | Allele frequencies | | | | | | | |
| --- | --- | --- | --- | --- | --- | --- | --- | --- |
|  | *O. nivara­­­­­­­­­­­­­­_________________* | | | | *O. rufipogon_______* | | | *O. meridionalis* |
|  | K1 | K2 | K3 | K4 | K5 | K6 | K7 | K8 |
| 238 | 0.000 | 0.000 | 0.056 | 0.000 | 0.000 | 0.045 | 0.000 | 0.000 |
| 239 | 0.000 | 0.000 | 0.000 | 0.000 | 0.058 | 0.000 | 0.000 | 0.000 |
| 240 | 0.000 | 0.000 | 0.000 | 0.000 | 0.080 | 0.062 | 0.000 | 0.000 |
| 242 | 0.000 | 0.000 | 0.000 | 0.000 | 0.000 | 0.004 | 0.012 | 0.000 |
| 245 | 0.000 | 0.000 | 0.000 | 0.000 | 0.000 | 0.000 | 0.070 | 0.000 |
| 274 | 0.000 | 0.000 | 0.000 | 0.000 | 0.022 | 0.000 | 0.000 | 0.000 |
|  |  |  |  |  |  |  |  |  |
| RM338 |  |  |  |  |  |  |  |  |
| 193 | 0.000 | 0.000 | 0.000 | 0.068 | 0.000 | 0.000 | 0.000 | 0.000 |
| 199 | 0.000 | 0.000 | 0.194 | 0.000 | 0.000 | 0.057 | 0.114 | 0.000 |
| 202 | 1.000 | 1.000 | 0.806 | 0.932 | 1.000 | 0.926 | 0.886 | 1.000 |
| 205 | 0.000 | 0.000 | 0.000 | 0.000 | 0.000 | 0.016 | 0.000 | 0.000 |
|  |  |  |  |  |  |  |  |  |
| RM408 |  |  |  |  |  |  |  |  |
| 130 | 0.022 | 0.000 | 0.000 | 0.000 | 0.000 | 0.000 | 0.000 | 0.000 |
| 134 | 0.000 | 0.000 | 0.014 | 0.137 | 0.000 | 0.061 | 0.000 | 0.375 |
| 136 | 0.196 | 0.829 | 0.000 | 0.164 | 0.109 | 0.246 | 0.080 | 0.000 |
| 138 | 0.130 | 0.000 | 0.806 | 0.336 | 0.094 | 0.066 | 0.091 | 0.625 |
| 140 | 0.489 | 0.171 | 0.056 | 0.205 | 0.493 | 0.508 | 0.830 | 0.000 |
| 142 | 0.000 | 0.000 | 0.097 | 0.041 | 0.116 | 0.045 | 0.000 | 0.000 |
| 144 | 0.098 | 0.000 | 0.000 | 0.068 | 0.101 | 0.070 | 0.000 | 0.000 |
| 146 | 0.065 | 0.000 | 0.028 | 0.041 | 0.051 | 0.000 | 0.000 | 0.000 |
| 148 | 0.000 | 0.000 | 0.000 | 0.007 | 0.000 | 0.000 | 0.000 | 0.000 |
| 150 | 0.000 | 0.000 | 0.000 | 0.000 | 0.000 | 0.004 | 0.000 | 0.000 |
| 154 | 0.000 | 0.000 | 0.000 | 0.000 | 0.036 | 0.000 | 0.000 | 0.000 |
|  |  |  |  |  |  |  |  |  |
| RM413 |  |  |  |  |  |  |  |  |
| 81 | 0.000 | 0.000 | 0.000 | 0.000 | 0.000 | 0.004 | 0.000 | 0.000 |
| 83 | 0.109 | 0.000 | 0.000 | 0.000 | 0.000 | 0.008 | 0.114 | 0.000 |
| 85 | 0.522 | 0.829 | 0.194 | 0.171 | 0.391 | 0.260 | 0.091 | 0.000 |
| 87 | 0.087 | 0.000 | 0.118 | 0.000 | 0.217 | 0.091 | 0.114 | 0.000 |
| 89 | 0.000 | 0.000 | 0.000 | 0.000 | 0.065 | 0.008 | 0.000 | 0.000 |
| 91 | 0.065 | 0.143 | 0.194 | 0.219 | 0.000 | 0.099 | 0.000 | 0.000 |
| 93 | 0.000 | 0.000 | 0.021 | 0.137 | 0.043 | 0.033 | 0.000 | 0.000 |
| 95 | 0.000 | 0.000 | 0.111 | 0.075 | 0.080 | 0.099 | 0.114 | 0.000 |
| 97 | 0.000 | 0.029 | 0.194 | 0.075 | 0.043 | 0.198 | 0.000 | 0.000 |
| 99 | 0.217 | 0.000 | 0.007 | 0.000 | 0.000 | 0.054 | 0.000 | 0.000 |

Table S3. continued

| Marker/ Allele size (bp) | Allele frequencies | | | | | | | |
| --- | --- | --- | --- | --- | --- | --- | --- | --- |
|  | *O. nivara­­­­­­­­­­­­­­_________________* | | | | *O. rufipogon_______* | | | *O. meridionalis* |
|  | K1 | K2 | K3 | K4 | K5 | K6 | K7 | K8 |
| 101 | 0.000 | 0.000 | 0.000 | 0.000 | 0.072 | 0.012 | 0.000 | 0.000 |
| 103 | 0.000 | 0.000 | 0.083 | 0.068 | 0.000 | 0.074 | 0.227 | 0.000 |
| 105 | 0.000 | 0.000 | 0.000 | 0.000 | 0.007 | 0.025 | 0.227 | 0.000 |
| 107 | 0.000 | 0.000 | 0.000 | 0.000 | 0.000 | 0.012 | 0.114 | 0.000 |
| **109** | 0.000 | 0.000 | 0.000 | 0.000 | 0.000 | 0.000 | 0.000 | 0.708 |
| **111** | 0.000 | 0.000 | 0.000 | 0.000 | 0.000 | 0.000 | 0.000 | 0.292 |
| 113 | 0.000 | 0.000 | 0.000 | 0.000 | 0.036 | 0.008 | 0.000 | 0.000 |
| 115 | 0.000 | 0.000 | 0.056 | 0.000 | 0.000 | 0.000 | 0.000 | 0.000 |
| 117 | 0.000 | 0.000 | 0.021 | 0.000 | 0.000 | 0.008 | 0.000 | 0.000 |
| 119 | 0.000 | 0.000 | 0.000 | 0.199 | 0.000 | 0.004 | 0.000 | 0.000 |
| 121 | 0.000 | 0.000 | 0.000 | 0.000 | 0.043 | 0.000 | 0.000 | 0.000 |
| 123 | 0.000 | 0.000 | 0.000 | 0.055 | 0.000 | 0.000 | 0.000 | 0.000 |
|  |  |  |  |  |  |  |  |  |
| RM431 |  |  |  |  |  |  |  |  |
| 248 | 0.000 | 0.000 | 0.000 | 0.055 | 0.000 | 0.000 | 0.000 | 0.000 |
| 250 | 0.000 | 0.000 | 0.000 | 0.000 | 0.000 | 0.008 | 0.000 | 0.000 |
| 252 | 0.109 | 0.714 | 0.014 | 0.000 | 0.058 | 0.008 | 0.000 | 0.167 |
| 254 | 0.109 | 0.000 | 0.007 | 0.000 | 0.043 | 0.102 | 0.000 | 0.000 |
| 256 | 0.022 | 0.029 | 0.000 | 0.000 | 0.130 | 0.090 | 0.364 | 0.000 |
| 258 | 0.011 | 0.000 | 0.056 | 0.068 | 0.319 | 0.238 | 0.114 | 0.000 |
| 260 | 0.000 | 0.029 | 0.000 | 0.041 | 0.116 | 0.283 | 0.341 | 0.000 |
| 262 | 0.652 | 0.000 | 0.215 | 0.068 | 0.181 | 0.004 | 0.000 | 0.208 |
| 264 | 0.022 | 0.000 | 0.153 | 0.151 | 0.094 | 0.078 | 0.045 | 0.208 |
| 266 | 0.000 | 0.229 | 0.194 | 0.260 | 0.014 | 0.090 | 0.000 | 0.083 |
| 268 | 0.054 | 0.000 | 0.194 | 0.226 | 0.007 | 0.082 | 0.102 | 0.000 |
| 270 | 0.022 | 0.000 | 0.069 | 0.055 | 0.022 | 0.016 | 0.000 | 0.000 |
| 272 | 0.000 | 0.000 | 0.000 | 0.068 | 0.014 | 0.000 | 0.000 | 0.125 |
| 276 | 0.000 | 0.000 | 0.083 | 0.000 | 0.000 | 0.000 | 0.023 | 0.000 |
| 278 | 0.000 | 0.000 | 0.000 | 0.000 | 0.000 | 0.000 | 0.000 | 0.208 |
| 280 | 0.000 | 0.000 | 0.014 | 0.007 | 0.000 | 0.000 | 0.000 | 0.000 |
| 282 | 0.000 | 0.000 | 0.000 | 0.000 | 0.000 | 0.000 | 0.011 | 0.000 |
|  |  |  |  |  |  |  |  |  |
| RM433 |  |  |  |  |  |  |  |  |
| 226 | 0.000 | 0.000 | 0.000 | 0.000 | 0.000 | 0.021 | 0.000 | 0.000 |
| 232 | 0.000 | 0.000 | 0.000 | 0.000 | 0.044 | 0.000 | 0.000 | 0.000 |
| 234 | 0.000 | 0.000 | 0.000 | 0.000 | 0.140 | 0.045 | 0.080 | 0.000 |
| 236 | 0.000 | 0.400 | 0.222 | 0.342 | 0.125 | 0.087 | 0.432 | 0.688 |

Table S3. continued

| Marker/ Allele size (bp) | Allele frequencies | | | | | | | |
| --- | --- | --- | --- | --- | --- | --- | --- | --- |
|  | *O. nivara­­­­­­­­­­­­­­_________________* | | | | *O. rufipogon_______* | | | *O. meridionalis* |
|  | K1 | K2 | K3 | K4 | K5 | K6 | K7 | K8 |
| 238 | 0.000 | 0.114 | 0.194 | 0.055 | 0.199 | 0.062 | 0.068 | 0.000 |
| 240 | 0.000 | 0.029 | 0.132 | 0.068 | 0.169 | 0.273 | 0.273 | 0.188 |
| 242 | 0.174 | 0.200 | 0.028 | 0.027 | 0.037 | 0.260 | 0.000 | 0.083 |
| 244 | 0.022 | 0.000 | 0.000 | 0.055 | 0.051 | 0.033 | 0.000 | 0.000 |
| 246 | 0.304 | 0.257 | 0.056 | 0.151 | 0.088 | 0.120 | 0.011 | 0.000 |
| 248 | 0.391 | 0.000 | 0.000 | 0.000 | 0.096 | 0.050 | 0.114 | 0.000 |
| 250 | 0.109 | 0.000 | 0.181 | 0.116 | 0.015 | 0.029 | 0.000 | 0.000 |
| 252 | 0.000 | 0.000 | 0.111 | 0.068 | 0.000 | 0.021 | 0.011 | 0.000 |
| 254 | 0.000 | 0.000 | 0.069 | 0.089 | 0.000 | 0.000 | 0.000 | 0.000 |
| 256 | 0.000 | 0.000 | 0.000 | 0.000 | 0.037 | 0.000 | 0.000 | 0.000 |
| 278 | 0.000 | 0.000 | 0.000 | 0.000 | 0.000 | 0.000 | 0.000 | 0.042 |
| 280 | 0.000 | 0.000 | 0.000 | 0.027 | 0.000 | 0.000 | 0.000 | 0.000 |
| 314 | 0.000 | 0.000 | 0.000 | 0.000 | 0.000 | 0.000 | 0.011 | 0.000 |
| 326 | 0.000 | 0.000 | 0.007 | 0.000 | 0.000 | 0.000 | 0.000 | 0.000 |
|  |  |  |  |  |  |  |  |  |
| RM447 |  |  |  |  |  |  |  |  |
| 122 | 0.000 | 0.057 | 0.000 | 0.014 | 0.000 | 0.017 | 0.000 | 1.000 |
| 125 | 0.000 | 0.000 | 0.000 | 0.014 | 0.022 | 0.000 | 0.000 | 0.000 |
| 128 | 0.000 | 0.629 | 0.183 | 0.090 | 0.179 | 0.054 | 0.114 | 0.000 |
| 131 | 0.000 | 0.000 | 0.000 | 0.042 | 0.007 | 0.004 | 0.159 | 0.000 |
| 134 | 0.283 | 0.000 | 0.042 | 0.063 | 0.366 | 0.017 | 0.193 | 0.000 |
| 137 | 0.000 | 0.000 | 0.014 | 0.167 | 0.142 | 0.308 | 0.000 | 0.000 |
| 140 | 0.000 | 0.000 | 0.056 | 0.181 | 0.000 | 0.250 | 0.250 | 0.000 |
| 143 | 0.000 | 0.143 | 0.528 | 0.250 | 0.037 | 0.125 | 0.193 | 0.000 |
| 146 | 0.000 | 0.171 | 0.000 | 0.000 | 0.187 | 0.133 | 0.091 | 0.000 |
| 149 | 0.522 | 0.000 | 0.014 | 0.125 | 0.000 | 0.063 | 0.000 | 0.000 |
| 152 | 0.152 | 0.000 | 0.070 | 0.000 | 0.000 | 0.004 | 0.000 | 0.000 |
| 155 | 0.000 | 0.000 | 0.000 | 0.000 | 0.022 | 0.000 | 0.000 | 0.000 |
| 158 | 0.000 | 0.000 | 0.092 | 0.056 | 0.000 | 0.000 | 0.000 | 0.000 |
| 161 | 0.000 | 0.000 | 0.000 | 0.000 | 0.000 | 0.025 | 0.000 | 0.000 |
| 164 | 0.043 | 0.000 | 0.000 | 0.000 | 0.037 | 0.000 | 0.000 | 0.000 |
|  |  |  |  |  |  |  |  |  |
| RM452 |  |  |  |  |  |  |  |  |
| 209 | 0.000 | 0.000 | 0.000 | 0.000 | 0.029 | 0.000 | 0.000 | 0.000 |
| 212 | 0.000 | 0.000 | 0.014 | 0.000 | 0.000 | 0.000 | 0.000 | 0.000 |
| 215 | 0.000 | 0.000 | 0.000 | 0.000 | 0.000 | 0.000 | 0.057 | 0.000 |
| 218 | 0.511 | 0.000 | 0.000 | 0.000 | 0.094 | 0.025 | 0.000 | 0.000 |

Table S3. continued

| Marker/ Allele size (bp) | Allele frequencies | | | | | | | |
| --- | --- | --- | --- | --- | --- | --- | --- | --- |
|  | *O. nivara­­­­­­­­­­­­­­_________________* | | | | *O. rufipogon_______* | | | *O. meridionalis* |
|  | K1 | K2 | K3 | K4 | K5 | K6 | K7 | K8 |
| 221 | 0.000 | 0.000 | 0.000 | 0.000 | 0.000 | 0.000 | 0.068 | 0.000 |
| 224 | 0.315 | 0.857 | 0.903 | 0.699 | 0.725 | 0.934 | 0.875 | 0.792 |
| 227 | 0.109 | 0.000 | 0.069 | 0.164 | 0.145 | 0.000 | 0.000 | 0.208 |
| 230 | 0.065 | 0.000 | 0.014 | 0.137 | 0.000 | 0.033 | 0.000 | 0.000 |
| 233 | 0.000 | 0.143 | 0.000 | 0.000 | 0.007 | 0.008 | 0.000 | 0.000 |
|  |  |  |  |  |  |  |  |  |
| RM455 |  |  |  |  |  |  |  |  |
| 145 | 0.891 | 1.000 | 0.986 | 0.986 | 0.746 | 0.775 | 0.989 | 1.000 |
| 149 | 0.109 | 0.000 | 0.014 | 0.000 | 0.254 | 0.217 | 0.011 | 0.000 |
| 153 | 0.000 | 0.000 | 0.000 | 0.014 | 0.000 | 0.008 | 0.000 | 0.000 |
|  |  |  |  |  |  |  |  |  |
| RM484 |  |  |  |  |  |  |  |  |
| 302 | 0.000 | 0.000 | 0.069 | 0.000 | 0.000 | 0.045 | 0.000 | 0.000 |
| 304 | 0.000 | 0.143 | 0.194 | 0.404 | 0.210 | 0.049 | 0.000 | 0.000 |
| 306 | 0.000 | 0.829 | 0.153 | 0.014 | 0.203 | 0.447 | 0.568 | 0.000 |
| 308 | 0.261 | 0.000 | 0.431 | 0.137 | 0.529 | 0.352 | 0.318 | 0.000 |
| 310 | 0.739 | 0.029 | 0.153 | 0.377 | 0.000 | 0.078 | 0.114 | 0.000 |
| 312 | 0.000 | 0.000 | 0.000 | 0.068 | 0.058 | 0.000 | 0.000 | 1.000 |
| 316 | 0.000 | 0.000 | 0.000 | 0.000 | 0.000 | 0.029 | 0.000 | 0.000 |
|  |  |  |  |  |  |  |  |  |
| RM495 |  |  |  |  |  |  |  |  |
| 165 | 0.467 | 0.500 | 0.972 | 0.932 | 0.594 | 0.803 | 0.523 | 1.000 |
| 171 | 0.000 | 0.000 | 0.000 | 0.000 | 0.080 | 0.102 | 0.432 | 0.000 |
| 174 | 0.533 | 0.500 | 0.007 | 0.062 | 0.232 | 0.020 | 0.011 | 0.000 |
| 177 | 0.000 | 0.000 | 0.021 | 0.007 | 0.094 | 0.074 | 0.034 | 0.000 |
|  |  |  |  |  |  |  |  |  |
| RM507 |  |  |  |  |  |  |  |  |
| 250 | 0.000 | 0.000 | 0.000 | 0.000 | 0.000 | 0.000 | 0.114 | 1.000 |
| 258 | 0.000 | 0.000 | 0.000 | 0.068 | 0.007 | 0.020 | 0.443 | 0.000 |
| 266 | 0.000 | 0.000 | 0.000 | 0.000 | 0.000 | 0.000 | 0.057 | 0.000 |
| 270 | 0.674 | 1.000 | 0.778 | 0.842 | 0.746 | 0.898 | 0.239 | 0.000 |
| 274 | 0.326 | 0.000 | 0.222 | 0.089 | 0.246 | 0.078 | 0.114 | 0.000 |
| 278 | 0.000 | 0.000 | 0.000 | 0.000 | 0.000 | 0.000 | 0.011 | 0.000 |
| 282 | 0.000 | 0.000 | 0.000 | 0.000 | 0.000 | 0.004 | 0.023 | 0.000 |
|  |  |  |  |  |  |  |  |  |
| RM536 |  |  |  |  |  |  |  |  |
| 235 | 0.109 | 0.000 | 0.028 | 0.000 | 0.000 | 0.000 | 0.000 | 0.000 |

Table S3. continued

| Marker/ Allele size (bp) | Allele frequencies | | | | | | | |
| --- | --- | --- | --- | --- | --- | --- | --- | --- |
|  | *O. nivara­­­­­­­­­­­­­­_________________* | | | | *O. rufipogon_______* | | | *O. meridionalis* |
|  | K1 | K2 | K3 | K4 | K5 | K6 | K7 | K8 |
| 237 | 0.435 | 0.000 | 0.000 | 0.260 | 0.072 | 0.037 | 0.000 | 0.000 |
| 239 | 0.109 | 0.000 | 0.042 | 0.000 | 0.000 | 0.000 | 0.000 | 0.000 |
| 241 | 0.000 | 0.000 | 0.070 | 0.000 | 0.145 | 0.123 | 0.364 | 0.000 |
| 243 | 0.109 | 0.000 | 0.493 | 0.164 | 0.667 | 0.328 | 0.500 | 0.750 |
| 245 | 0.022 | 0.886 | 0.225 | 0.507 | 0.080 | 0.504 | 0.136 | 0.250 |
| 247 | 0.000 | 0.000 | 0.056 | 0.000 | 0.000 | 0.000 | 0.000 | 0.000 |
| 249 | 0.000 | 0.000 | 0.014 | 0.068 | 0.007 | 0.008 | 0.000 | 0.000 |
| 251 | 0.174 | 0.114 | 0.070 | 0.000 | 0.029 | 0.000 | 0.000 | 0.000 |
| 253 | 0.043 | 0.000 | 0.000 | 0.000 | 0.000 | 0.000 | 0.000 | 0.000 |
